# The Effects of Familiarisation on Countermovement Jumps with Handheld Dumbbell Accentuated Eccentric Loading in Youth Athletes

**DOI:** 10.1002/ejsc.70033

**Published:** 2025-08-12

**Authors:** Thomas E. Bright, Jason Lake, Matthew J. Handford, Nicola Theis, Peter Mundy, Jonathan D. Hughes

**Affiliations:** ^1^ Youth Physical Development Centre, School of Sport and Health Sciences Cardiff Metropolitan University Cardiff UK; ^2^ School of Sport, Exercise and Rehabilitation Plymouth Marjon University Plymouth UK; ^3^ Institute of Sport, Nursing, and Allied Health University of Chichester Chichester UK; ^4^ U7 Performance Exeter UK; ^5^ School of Sport and Exercise University of Gloucestershire Gloucester UK; ^6^ Hawkin Dynamics Inc. Westbrook Maine USA

**Keywords:** eccentric training, jumping, learning effect, long‐term athlete development, statistical parametric mapping

## Abstract

This study was used to investigate the effects of familiarisation on a countermovement jump (CMJ) performed with handheld dumbbell accentuated eccentric loading (AEL) at 20% of body mass (CMJ_AEL20_). Twenty‐seven adolescent males performed CMJ_AEL20_ on three separate occasions. Statistical parametric mapping (SPM) detected significant differences in normalised force‐time data between session one and two (50%–95% of movement time), two and three (47%–48%) and one and three (66%–96%), but not in velocity‐ or displacement‐time data. Propulsion mean vertical ground reaction force (vGRF) had excellent reliability (CV% upper CI_95_ = 5.12–9.33; ICC lower CI_95_ = 0.99), whereas jump height exhibited good relative reliability (ICC lower CI_95_ ≥ 0.94) but moderate to poor absolute reliability (CV% upper CI_95_ = 6.72–15.36). Unweighting time and braking time showed moderate to poor reliability (CV% upper CI_95_ = 14.22–37.06; ICC lower CI_95_ = 0.46–0.89). Mean bias between sessions was ≤ 10% for all variables according to repeated measures Bland‐Altman analysis; however, fixed bias was observed in braking mean vGRF and propulsion mean velocity. Jump height, braking mean vGRF, propulsion mean vGRF and propulsion mean velocity exhibited good to acceptable limits of agreement (LOA; ≤ 20%), whereas all other variables were classified as ‘poor’ (> 20%). Proportional bias was identified in unweighting vGRF%, braking mean vGRF and braking mean velocity. These findings suggest that although more than three familiarisation sessions may be required for unweighting and braking CMJ_AEL20_ variables, reliable propulsion data, including jump height, were observed from session one.

## Highlights

- Significant differences in normalised force‐time data were observed between three CMJ_AEL20_ familiarisation sessions, particularly after the dumbbells' release point, indicating a potential learning effect.
- Propulsion phase variables were reliable from the first session; however, unweighting and braking phase variables showed poor to moderate reliability, suggesting more than three familiarisation sessions are needed for these phases of the CMJ_AEL_.
- Braking phase time demonstrated the poorest reliability between‐ and within‐sessions, likely because of inconsistent dumbbell release timing.
- Future studies should investigate phase‐specific verbal cues to stabilise movement execution and enhance performance during CMJ_AEL_.

## 1 Introduction

There is a well‐established body of evidence supporting the prioritisation of plyometric‐jump training (PJT) to enhance rapid force production characteristics in youth athletes (Sammoud et al. 2022; Ramirez‐Campillo et al. 2023). This is because the observed effects on physical and sports performance (e.g., vertical jumping, standing long jump and sprinting with and without ball) can be greater than that associated with growth and maturation alone (Ramirez‐Campillo et al. 2023; Asadi et al. 2018). As a result, there are a growing number of studies investigating the effects of applying external load during PJT in this population (Kobal et al. 2017; Loturco et al. 2016; Rosas et al. 2016). A potential issue with this approach is that while it may promote an increase in force, movement velocity may decrease (Mundy et al. 2017; Swinton et al. 2012). However, if the external load could be used to selectively increase eccentric (termed braking herein) force production without a simultaneous reduction in propulsion phase velocity, it may be possible to realise an increase in jump height (Laffaye and Wagner 2013; Nishiumi et al. 2023; Barker et al. 2018). Furthermore, these training methods could be used to target braking phase characteristics, which would be particularly beneficial when considering the biomechanical demands of change of direction and deceleration tasks in sport (McBurnie and Dos’Santos 2022; McBurnie et al. 2022; Harper et al. 2022).

Accentuated eccentric loading (AEL) is a form of movement manipulation that has been suggested to acutely enhance force and velocity output during PJT (Handford et al. 2021; Wagle et al. 2017; J. Merrigan et al. 2022). It is most commonly applied to a countermovement jump (CMJ_AEL_) or drop jump and requires an external load to be added to the body during the downward movement before being released at or shortly before the transition to upward movement (Handford et al. 2021). Some researchers have observed significant improvements in jump height, peak power, force and propulsive velocity when using this approach with the application of handheld free weights or elastic resistance (Lloyd et al. 2022; Sheppard et al. 2007; Aboodarda et al. 2013; Godwin et al. 2021; L. Bridgeman et al. 2016). In contrast, when the movement is more constrained (e.g., custom‐built device, barbell or trap bars with weight releasers), an increase in braking phase characteristics but reductions in jump height and reactive strength index have been documented in the literature (Lloyd et al. 2022; Su et al. 2023; Moore et al. 2007; Taber et al. 2023; Aboodarda et al. 2014). One possible explanation for these reported differences could be the magnitude of AEL applied. It has been noted that loads around 15%–30% of body mass tend to elicit the most consistent improvements in braking and propulsion performance (Lloyd et al. 2022; Aboodarda et al. 2013; Godwin et al. 2021; Aboodarda et al. 2014). The benefits, particularly during the propulsion phase, also appear most pronounced in individuals with higher levels of training experience and, in turn, relative strength (Sheppard et al. 2007; Godwin et al. 2021; Taber et al. 2023). Based upon these collective findings, it can be suggested that the acute responses to CMJ_AEL_ are dependent on the choice of equipment, the magnitude of load, participant characteristics and, importantly, the relative intensity of execution (i.e., percentage of maximal effort), as intent appears critical for eliciting favourable changes in PJT (Salles et al. 2011).

It is important to note that only one study to date has investigated the application of AEL during PJT with youth athletes. Specifically, Lloyd et al. (2022) reported a significant acute increase in jump height and braking and propulsion impulse after using AEL applied with the use of handheld dumbbells at 15% of body mass during a drop jump; however, these changes were accompanied by longer ground contact times and a reduction in spring‐like behaviour (Lloyd et al. 2022). It is possible that the participants in this study did not possess an adequate level of strength to maintain technical competency and prevent an extended time delay between braking and propulsion actions, even without AEL (J. Merrigan et al. 2022; J. J. Merrigan et al. 2021). Nonetheless, it is more likely that the use of a mechanically demanding exercise (i.e., drop jump), when coupled with AEL, exceeded the participants' capacity to utilise the increase in braking impulse experienced (Gillen et al. 2021, 2019). Therefore, applying this loading scheme to a technically and physically easier movement to execute in the CMJ may not only have been more appropriate but could also facilitate the application of a greater load. This possibility warrants consideration in future research.

Because CMJ_AEL_ requires precise execution in order to release the external load without effecting the natural characteristics of the movement (Bright et al. 2024), it is important to consider if familiarisation is necessary prior to implementing in youth strength and conditioning (S&C) programmes. To the best of our knowledge, only a few investigations have focused on the impact of familiarisation on PJT (Cabarkapa et al. 2024; Moir et al. 2004, 2005; Nibali et al. 2015). For example, Moir et al. (2005) examined the number of familiarisation sessions required before an acceptable level of reliability associated with unloaded and loaded CMJ performance in physically active males was met. The authors showed that good reliability (CV% ≤ 4.2; ICC ≥ 0.75) can be achieved in the aforementioned tests without the implementation of familiarisation sessions (Moir et al. 2005). Similar findings were observed by Nibali et al. (2015), indicating that both male and female athletes do not require familiarisation trials before CMJ assessment, regardless of the level of play (i.e., high school, college and professional). More recently, researchers have found that the reliability of CMJ variables did not change over four familiarisation sessions in college‐aged males (Cabarkapa et al. 2024). However, Vrbik et al. (2017) reported that elementary school children (age = 10.8 years) significantly improved their CMJ and standing long jump performance from the first to penultimate and ultimate testing sessions, respectively (i.e., five total testing sessions). Taken together, it is likely that the importance of familiarisation depends on exercise complexity and intensity, as well as participant age, maturity and previous test experience (Gillen et al. 2018; Bright et al. 2023). It is therefore reasonable to assume that: (1) reliable unloaded CMJ performance is a prerequisite for introducing CMJ_AEL_ in younger, less mature individuals only; (2) CMJ_AEL_ is best reserved for older adolescents (i.e., post‐peak height velocity [PHV]) and (3) repeated trials and sessions will be required to ensure adequate familiarisation.

The primary aim of this study was to investigate the differences in CMJ_AEL_ normalised force‐, velocity‐ and displacement‐time characteristics between three separate testing sessions. A secondary purpose of this study was to examine the reliability, agreement and differences between discrete CMJ_AEL_ variables. Despite limited research on CMJ_AEL_, we hypothesised that: (1) there would be significant differences in normalised force‐time characteristics between session one, two and three, owing to the complexity of the movement and (2) there would be no significant differences between sessions in normalised velocity‐ and displacement‐time signals or discrete performance variables. Based upon a recent investigation (Bright et al. 2024), it was also hypothesised that jump height, braking and propulsion force would demonstrate acceptable reliability within‐ and between‐sessions; however, countermovement depth and braking and propulsion time and velocity were expected to display reduced reliability and agreement because of the inherent variability associated with these measures.

## 2 Methods

### 2.1 Participants

A convenience sample of 27 male adolescent participants (age: 15.0 ± 0.9 years; height: 1.72 ± 0.60 m; body mass: 62.2 ± 8.2 kg; percentage of predicted adult height: 96.3 ± 2.7) volunteered to participate in this study. Participants were a mixture of defenders, midfielders and attackers and were categorised as trained (i.e., local‐level representation, regularly training ∼3 times per week, primary sport is soccer and training with a purpose to compete but limited skill development) according to a classification framework (McKay et al. 2021). All were free from injury and were involved in regular sport training and physical education‐based activity programmes, including S&C, a minimum of once a week. This included a minimum of 12 months' exposure to weekly strength training that primarily involved compound movements (e.g., back squat, deadlift and bench press). Participants also had at least 6 months of experience performing the CMJ as part of training and routine testing. Written parental consent, participant assent and a standardised physical activity readiness questionnaire were completed prior to any child being involved in the study. Ethical approval for the research was granted by the University Research Ethics Committee in accordance with the Declaration of Helsinki (REC.22.32.1a).

### 2.2 Experimental Protocol

The biological maturity status of each participant was estimated using the Khamis–Roche percentage of the predicted adult height method (Khamis and Roche 1994). Participant's standing height, body mass, chronological age at observation and mid‐parental standing height were used to apply this method (Khamis and Roche 1994). Parental height was collected by a member of the research team (TB), or where collection was not possible, it was self‐reported by the parents and subsequently adjusted for overestimation (Epstein et al. 1995). The percentage of predicted adult height was calculated by dividing participant's current height by their predicted adult height and multiplying by 100 (Khamis and Roche 1994).

Participants completed a standardised 10‐min dynamic warm‐up at the start of each session. This began with 5 min of stationary cycling at a self‐selected pace and was followed by 10 bodyweight squats, reverse lunges and jump squats. Participants were familiarised with the CMJ_AEL_ in session one, which involved practicing the movement until the lead researcher was satisfied with the technical execution. A load of 20% of body mass was selected based on prior research using 15% body mass during a more technically demanding drop jump task (Lloyd et al. 2022). Given the lower mechanical complexity of the CMJ (Gillen et al. 2021, 2019), it was reasoned that a slightly higher load could be tolerated without disrupting movement mechanics. This load was also piloted to ensure it could be executed safely and consistently. All participants were able to perform the movement with no major technical issues within six repetitions (mean = 4.56 repetitions; range = 4–6 repetitions).

Data collection in session one, two and three involved three trials of CMJ_AEL20_, separated by approximately 1 min of rest. Participants were provided with the following instructions prior to each trial: ‘*perform the countermovement at your maximum comfortable speed, release the dumbbells as close to your lowest position as possible before moving upward and continue to jump as fast and as high as possible*’. These instructions were chosen to prevent participants from purposefully releasing the dumbbells significantly before or after their lowest position and subsequently altering the fluidity of the movement. After releasing the dumbbells, participants were cued to return their arms to the akimbo position for the remainder of the jump and landing. Emphasis was also placed on returning to an upright still position as quickly as possible (Bright et al. 2024; Wade et al. 2022).

### 2.3 Data Analysis

Raw vertical ground reaction force (vGRF) data from two force platforms (Kistler Type 9286B, Kistler Instruments, Winterthur, Switzerland; 1000 Hz) were summed to represent the vGRF acting at the whole‐body centre of mass (CoM). Based on a residual analysis, the vGRF data were smoothed using a fourth‐order, bidirectional, low‐pass Butterworth digital filter with a cut‐off frequency of 50 Hz (Harry et al. 2022). The vGRF signal was filtered after phases (unweighting, braking and propulsion phases; described below) were identified to ensure that it did not affect their location (Street et al. 2001).

System weight, representing bodyweight + dumbbell weight, was obtained by averaging 1 s of the vGRF signal at the start of the jump (forward integration; FI). The vGRF signal was flipped using MATLABs ‘flipud’ function such that the first recorded data point became the last data point in the time history (Bright et al. 2024). Bodyweight was subsequently calculated from 1 s of the postlanding period where participants were instructed to return to an upright and motionless position as quickly as possible (backward integration; BI) (Bright et al. 2024). These periods were used to ensure an initial velocity of 0 m·s^−1^ and to identify the vGRF mean and SD which was subsequently used to detect the start of the movement. For FI and BI, if the mean vGRF between the end of the stance phase and the lowest position was greater than the stance mean + 5 × SD, then we searched for the first value greater than the stance mean + 5 × SD (preload strategy); if not, we searched for the first vGRF value less than stance mean − 5 × SD (unload strategy). Net vGRF (vGRF − system weight) was divided by the system mass to calculate CoM acceleration. The CoM acceleration was integrated using the trapezoidal rule to obtain CoM velocity, which was then integrated to calculate CoM displacement (Owen et al. 2014). Finally, the BI CoM acceleration, velocity and displacement signals were flipped to match the original direction of the vGRF signal.

The difference between FI and BI signals was calculated, and the first instance at which the difference was equal to zero (i.e., signal intersection point) was used to identify the point in which the dumbbells were released (Bright et al. 2024). This was chosen as it represents the instant at which the two masses (e.g., system mass and body mass) became equal as they transitioned to take each other's magnitude.

The unweighting phase was calculated as the duration between the start of movement and peak negative CoM velocity in the FI signal. The braking phase began one sample after this point, ending at the first occurrence of a CoM velocity of ≥ 0 m·s^−1^, using the BI velocity signal (McMahon et al. 2018). This also coincided with the beginning of the propulsion phase, which ended at take‐off (identified using a 10 N threshold). Likewise, ground contact was defined using a 10 N threshold which was based on the analysis of the residual vGRF when the force platforms were unloaded.

Jump height (takeoff velocity^2^ ÷ 2 × *g* [acceleration of gravity]), countermovement depth, phase times, mean force and velocity, as well as unweighting minimum force as a percentage of body weight were then calculated. Force‐, velocity‐ and displacement‐time signals were time‐normalised to 500 data points representing 0%–100% of the movement from the start of the countermovement to take‐off. This was achieved by changing the time delta between the original samples (e.g., original number of samples/500) and subsequently resampling the data (Cormie et al. 2009).

### 2.4 Statistical Analysis

A two‐way random effects model (absolute agreement, average measures) and intraclass correlation coefficient (ICC), along with upper and lower 95% confidence intervals (CI_95_), were used to determine between‐ and within‐session relative reliability. Based on the lower CI_95_ of the ICC estimate, values were interpreted as < 0.5, poor; 0.5–0.75, moderate, 0.75–0.90, good and > 0.90, excellent (Koo and Li 2016). Absolute between‐ and within‐session reliability was assessed using the coefficient of variation (CV%), which was computed via the mean square root approach. To provide a qualitative scale in line with the ICC estimates, CV% thresholds of < 5%, 5%–10%, 10%–15% and > 15% (based on the upper CI_95_) were considered to represent excellent, good, moderate and poor reliability, respectively.

The use of ICC and CV% measurements alone to quantify reliability is frequently scrutinised because neither adequately account for systematic bias and random error (Warneke et al. 2025; Atkinson and Nevill 1998). To overcome this, we also calculated Bland–Altman limits of agreement (LOA) for repeated measurements (Bland and Altman 2007). Absolute values were converted to a percentage and three a prior categories were defined for the bias and LOA: (1) good—suitable for training purposes (≤ 10%), (2) acceptable—suitable for training purposes, but consideration needs to be given to execution (> 10 to ≤ 20%) and (3) poor—not suitable for training purposes, further familiarisation required (> 20%). These thresholds were defined based on recent reliability data (Bright et al. 2023) while considering that the purpose of this study was to assess familiarisation for training rather than for testing and monitoring. To further supplement this analysis, fixed bias was assessed using one‐sample *t*‐tests and proportional bias was evaluated using linear regression between mean scores and the corresponding differences across the three testing sessions.

A one‐way repeated measures analysis of variances (ANOVA) was conducted to compare the differences between dependent variables. The Greenhouse–Geisser correction was used when the Mauchly's sphericity test was violated and pairwise differences were identified using Bonferroni post hoc corrections. Effect sizes were calculated using Hedges' *g* method, providing a measure of the magnitude of the differences between sessions. These were interpreted as trivial (≤ 0.19), small (0.20–0.49), moderate (0.50–0.79) or large (≥ 0.80) (Cohen 2009).

Statistical parametric mapping (SPM) was used to compare changes in normalised force‐, velocity‐ and displacement‐time data between session one, session two and session three using the open‐source ‘spm1d’ package for Matlab (R2022b; The Mathworks Inc., Natick, MA, USA) (located at http://www.spm1d.org/). An SPM one‐way repeated measures ANOVA was conducted to compare signals between sessions with *α* = 0.05. Where significant effects (*p* < 0.05) were observed, post hoc analysis using an SPM paired samples *t*‐test and Bonferroni correction were used to compare between sessions.

## 3 Results

Descriptive data are presented in Table 1 and the between‐session reliability statistics are presented in Table 2. Unweighting vGRF, braking mean vGRF, propulsion mean vGRF and propulsion mean velocity demonstrated excellent absolute reliability across all session comparisons, whereas relative reliability was good to excellent. Jump height, countermovement depth, unweighting time, propulsion time and braking mean velocity displayed good absolute reliability and moderate to excellent relative reliability in all session comparisons. Braking time displayed moderate absolute reliability in session two versus three and one versus three, but poor absolute reliability in session one versus two. Braking time relative reliability was moderate in session one versus two and one versus three and poor in session two versus three.

**TABLE 1:** Mean and SD data for all CMJ_AEL20_ measured variables in session one, two and three.

Variables	Session one		Session two		Session three	
	Mean	SD	Mean	SD	Mean	SD
Jump height (m)	0.29	0.06	0.29	0.06	0.29	0.06
Countermovement depth (m)	−0.33	0.05	−0.33	0.05	−0.32	0.06
Unweighting time (s)	0.51	0.12	0.50	0.13	0.49	0.11
Braking time (s)	0.19	0.05	0.18	0.05	0.19	0.05
Propulsion phase time (s)	0.32	0.05	0.32	0.05	0.33	0.06
Unweighting vGRF (% BW)	45.29	13.51	45.46	12.37	46.14	16.67
Braking mean vGRF (N)	963.53	153.92	988.39	152.92	982.39	167.37
Propulsion mean vGRF (N)	1070.94	189.41	1079.15	174.72	1068.96	193.74
Braking mean velocity (m·s^−1^)	0.63	0.15	0.65	0.12	0.64	0.14
Propulsion mean velocity (m·s^−1^)	1.39	0.17	1.40	0.17	1.36	0.20

Abbreviations: BW, bodyweight; SD, standard deviation; vGRF, vertical ground reaction force.

**TABLE 2:** Between‐session reliability statistics for all CMJ_AEL20_ measured variables.

Variables	Session one versus two		Session two versus three		Session one versus three	
	CV% (CI_95_)	ICC (CI_95_)	CV% (CI_95_)	ICC (CI_95_)	CV% (CI_95_)	ICC (CI_95_)
Jump height (m)	6.13 (5.15, 7.58)	0.97 (0.95, 0.99)	5.85 (4.91, 7.22)	0.98 (0.96, 0.99)	6.15 (4.91, 7.22)	0.97 (0.94, 0.98)
Countermovement depth (m)	6.24 (5.24, 7.71)	0.92 (0.87, 0.96)	6.69 (5.62, 8.26)	0.92 (0.86, 0.96)	6.37 (5.62, 8.26)	0.89 (0.82, 0.95)
Unweighting time (s)	6.90 (5.80, 8.53)	0.87 (0.78, 0.94)	7.37 (6.19, 9.11)	0.77 (0.60, 0.88)	7.24 (6.19, 9.11)	0.77 (0.60, 0.88)
Braking time (s)	12.44 (10.44, 15.42)	0.83 (0.70, 0.91)	11.16 (9.36, 13.82)	0.69 (0.46, 0.84)	12.08 (9.36, 13.82)	0.72 (0.52, 0.86)
Propulsion phase time (s)	5.75 (4.83, 7.11)	0.92 (0.86, 0.96)	5.70 (4.79, 7.04)	0.92 (0.86, 0.96)	5.57 (4.79, 7.04)	0.88 (0.79, 0.94)
Unweighting vGRF (% BW)	0.77 (0.65, 0.95)	0.94 (0.89, 0.97)	0.76 (0.64, 0.93)	0.93 (0.88, 0.96)	0.78 (0.64, 0.93)	0.91 (0.84, 0.95)
Braking mean vGRF (N)	0.09 (0.08, 0.11)	0.98 (0.96, 0.99)	0.08 (0.07, 0.10)	0.94 (0.89, 0.97)	0.09 (0.07, 0.10)	0.93 (0.88, 0.97)
Propulsion mean vGRF (N)	0.07 (0.06, 0.09)	0.99 (0.98, 0.99)	0.07 (0.06, 0.09)	0.99 (0.98, 1.00)	0.07 (0.06, 0.09)	0.99 (0.98, 0.99)
Braking mean velocity (m·s^−1^)	5.69 (4.78, 7.03)	0.93 (0.89, 0.97)	5.41 (4.55, 6.68)	0.91 (0.84, 0.95)	6.17 (4.55, 6.68)	0.88 (0.79, 0.94)
Propulsion mean velocity (m·s^−1^)	2.29 (1.92, 2.83)	0.97 (0.95, 0.98)	2.39 (2.01, 2.95)	0.89 (0.82, 0.95)	2.56 (2.01, 2.95)	0.88 (0.79, 0.94)

Abbreviations: BW, bodyweight; CI_95_, 95% confidence intervals; CV%, coefficient of variation percentage; ICC, intraclass correlation coefficient; vGRF, vertical ground reaction force.

The within‐session reliability data for all variables are presented in Table 3. Unweighting vGRF, braking mean vGRF and propulsion mean vGRF demonstrated excellent absolute reliability and moderate to excellent relative reliability within all sessions. Propulsion mean velocity absolute reliability was excellent within session one and two and good in session three, whereas relative reliability was good to excellent within session one and two and poor in session three. Braking mean velocity absolute reliability was good within session two and moderate within session one and three and relative reliability was moderate to good within all sessions. Jump height, countermovement depth and propulsion phase time absolute reliability was moderate and relative reliability was moderate to excellent within all sessions. Unweighting time absolute reliability was moderate in session one and two and poor in session three, whereas relative reliability was moderate in session one and poor in session two and three. Braking time absolute and relative reliability was poor within all sessions.

**TABLE 3:** Within‐session reliability statistics for all CMJ_AEL20_ measured variables.

Variables	Session one		Session two		Session three	
	CV% (CI_95_)	ICC (CI_95_)	CV% (CI_95_)	ICC (CI_95_)	CV% (CI_95_)	ICC (CI_95_)
Jump height (m)	9.09 (7.14, 12.49)	0.96 (0.92, 0.98)	8.23 (6.47, 11.31)	0.97 (0.93, 0.98)	8.30 (6.50, 11.50)	0.97 (0.95, 0.99)
Countermovement depth (m)	8.33 (6.55, 11.45)	0.92 (0.86, 0.96)	9.28 (7.29, 12.76)	0.90 (0.82, 0.95)	9.63 (7.54, 13.34)	0.82 (0.66, 0.91)
Unweighting time (s)	9.56 (7.52, 13.15)	0.83 (0.67, 0.91)	9.96 (7.83, 13.70)	0.73 (0.48, 0.87)	10.88 (8.51, 15.09)	0.41 (0.13, 0.72)
Braking time (s)	18.77 (14.68, 26.08)	0.68 (0.40, 0.85)	16.34 (12.80, 22.62)	0.68 (0.41, 0.85)	15.20 (11.87, 21.18)	0.54 (0.11, 0.78)
Propulsion phase time (s)	7.95 (6.25, 10.92)	0.92 (0.85, 0.96)	8.32 (6.54, 11.43)	0.93 (0.87, 0.97)	7.79 (6.11, 10.79)	0.87 (0.76, 0.94)
Unweighting vGRF (% BW)	1.12 (0.88, 1.53)	0.91 (0.82, 0.96)	1.06 (0.84, 1.45)	0.91 (0.82, 0.95)	1.08 (0.85, 1.49)	0.86 (0.74, 0.93)
Braking mean vGRF (N)	0.14 (0.11, 0.19)	0.96 (0.93, 0.98)	0.12 (0.10, 0.17)	0.97 (0.95, 0.99)	0.12 (0.09, 0.16)	0.80 (0.62, 0.90)
Propulsion mean vGRF (N)	0.10 (0.08, 0.14)	0.99 (0.98, 0.99)	0.10 (0.08, 0.14)	0.99 (0.98, 0.99)	0.10 (0.08, 0.13)	0.99 (0.98, 1.00)
Braking mean velocity (m·s^−1^)	9.08 (7.14, 12.48)	0.86 (0.74, 0.93)	6.86 (5.40, 9.42)	0.92 (0.86, 0.96)	8.37 (6.55, 11.58)	0.77 (0.56, 0.89)
Propulsion mean velocity (m·s^−1^)	3.49 (2.75, 4.78)	0.94 (0.88, 0.97)	2.97 (2.34, 4.07)	0.97 (0.95, 0.99)	3.74 (2.94, 5.17)	0.71 (0.44, 0.86)

Abbreviations: BW, bodyweight; CI_95_, 95% confidence intervals; CV%, coefficient of variation percentage; ICC, intraclass correlation coefficient; vGRF, vertical ground reaction force.

**FIGURE 1:**
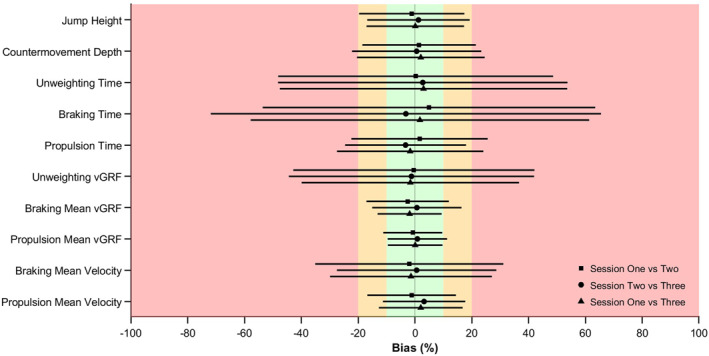
Percentage bias and LOA between session one versus two, two versus three and one versus three for CMJ_AEL20_ variables. Green, amber and red shaded areas represent good, acceptable and poor familiarisation has been achieved, respectively. The original bias, LOA and 95% confidence intervals are presented in Supporting Information S1: Appendix A alongside associated fixed and proportional bias data.

Mean bias between sessions for all CMJ_AEL_ variables was ≤ 10% (Figure 1). The LOA was ‘good’ to ‘acceptable’ (≤ 20%) for jump height, braking mean vGRF, propulsion mean vGRF and propulsion mean velocity. All remaining LOAs were > 20% and were therefore considered ‘poor’.

Fixed bias was identified in two comparisons: braking mean vGRF (session one vs. two; *p* = 0.010) and propulsion mean velocity (session two vs. three; *p* = 0.003), despite both variables displaying ‘good’ mean bias based on the predefined thresholds (≤ 10%). Proportional bias was observed in unweighting vGRF% (session two vs. three and one vs. three: *β* = −0.33 and −0.26; adjusted *R* ^2^ = 0.17 and 0.05; *p* < 0.05), braking mean vGRF (session two vs. three: *β* = −0.09; adjusted *R* ^2^ = 0.04; *p* = 0.047) and braking mean velocity (session one vs. two: *β* = 0.22; adjusted *R* ^2^ = 0.06; *p* = 0.014). The Bland–Altman plots are presented alongside their respective bias, LOA and 95% confidence intervals in Supporting Information S1: Appendix A.

Between‐session mean differences and Hedge's *g* effect sizes are presented in Table 4. Although there were no significant between‐session differences in any of the dependent variables (*p* ≥ 0.085), a small effect size was noted between session two and three (*g* = −0.20).

**TABLE 4:** Mean differences and Hedge's *g* effect size estimates between sessions for all CMJ_AEL20_ measured variables.

Variables	Session one versus two		Session two versus three		Session one versus three	
	Δ (CI_95_)	Hedges *g* (CI_95_)	Δ (CI_95_)	Hedges *g* (CI_95_)	Δ (CI_95_)	Hedges *g* (CI_95_)
Jump height (m)	0.00 (−0.01, 0.01)	−0.05 (−0.59, 0.48)	0.00 (−0.01, 0.01)	0.06 (−0.47, 0.60)	0.00 (−0.01, 0.01)	0.01 (−0.53, 0.54)
Countermovement depth (m)	0.00 (−0.02, 0.01)	−0.09 (−0.63, 0.44)	0.00 (−0.01, 0.01)	−0.03 (−0.57, 0.50)	−0.01 (−0.02, 0.01)	−0.12 (−0.65, 0.42)
Unweighting time (s)	0.00 (−0.03, 0.03)	0.01 (−0.52, 0.54)	0.01 (−0.01, 0.04)	0.11 (−0.42, 0.65)	0.02 (−0.02, 0.05)	0.13 (−0.41, 0.66)
Braking time (s)	0.01 (−0.01, 0.02)	0.18 (−0.35, 0.71)	−0.01 (−0.02, 0.01)	−0.11 (−0.64, 0.43)	0.00 (−0.01, 0.02)	0.08 (−0.45, 0.61)
Propulsion phase time (s)	0.01 (−0.01, 0.02)	0.10 (−0.43, 0.64)	−0.01 (−0.02, 0.00)	−0.20 (−0.73, 0.34)	−0.01 (−0.02, 0.01)	−0.10 (−0.63, 0.43)
Unweighting vGRF (% BW)	−0.18 (−3.36, 3.00)	−0.01 (−0.55, 0.52)	−0.68 (−3.86, 2.50)	−0.05 (−0.58, 0.49)	−0.86 (−5.61, 3.89)	−0.06 (−0.59, 0.48)
Braking mean vGRF (N)	−24.86 (−43.45, −6.27)	−0.16 (−0.69, 0.37)	6.00 (−12.59, 24.59)	0.04 (−0.50, 0.57)	−18.86 (−47.40, 9.68)	−0.12 (−0.65, 0.42)
Propulsion mean vGRF (N)	−8.21 (−29.58, 13.16)	−0.04 (−0.58, 0.49)	10.19 (−11.19, 31.56)	0.05 (−0.48, 0.59)	1.97 (−29.07, 33.02)	0.01 (−0.52, 0.54)
Braking mean velocity (m·s^−1^)	−0.01 (−0.04, 0.02)	−0.09 (−0.63, 0.44)	0.00 (−0.03, 0.03)	0.02 (−0.51, 0.55)	−0.01 (−0.05, 0.03)	−0.07 (−0.60, 0.46)
Propulsion mean velocity (m·s^−1^)	−0.02 (−0.06, 0.03)	−0.09 (−0.62, 0.44)	0.04 (0.00, 0.09)	0.23 (−0.30, 0.77)	0.03 (−0.02, 0.08)	0.15 (−0.39, 0.68)

*Note:* Positive effect size estimates indicate an increase relative to the previous session. Negative effect size estimates indicate a decrease relative to the previous session.

Abbreviations: BW, bodyweight; CI_95_, 95% confidence intervals; vGRF, vertical ground reaction force.

**FIGURE 2:**
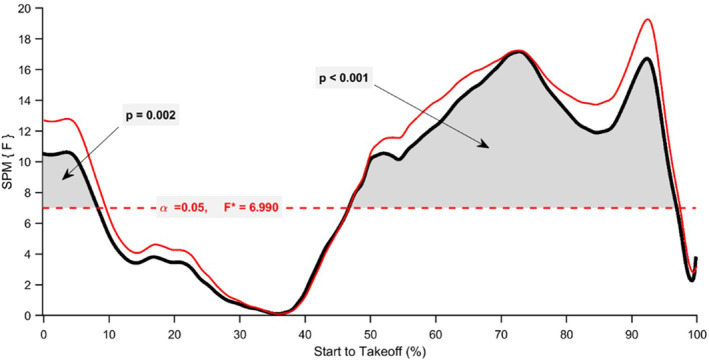
SPM repeated measures ANOVA during CMJ_AEL20_ comparing normalised force‐time signals between session one, session two and session three.

**FIGURE 3:**
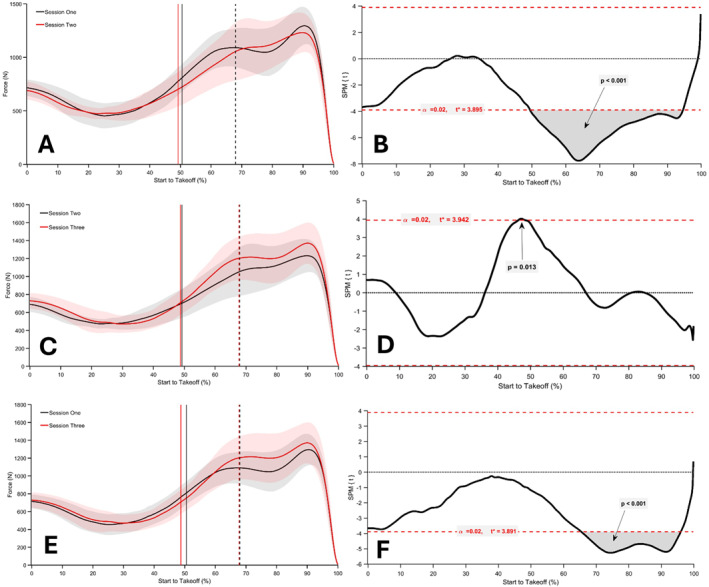
Mean and SD normalised force‐time signals for CMJ_AEL20_ in session one versus two (A), session two versus three (C) and session one versus three (E), respectively. In A, C and E, the solid vertical lines represent the dumbbell release point, and the dashed vertical lines represent the lowest position. SPM paired *t*‐test results for each session comparison are illustrated in B, D and F.

**FIGURE 4:**
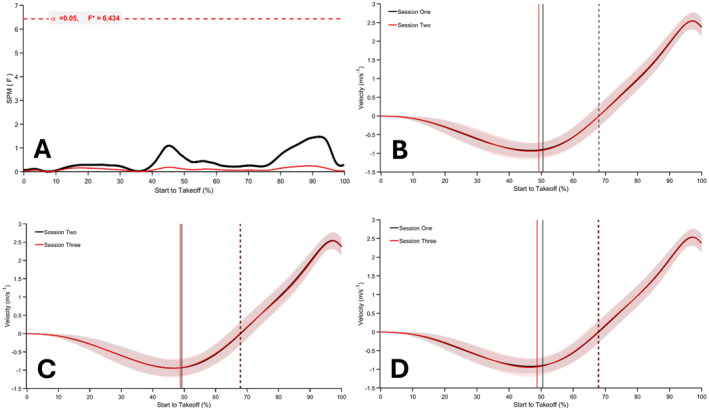
SPM repeated measures ANOVA during CMJ_AEL20_ comparing normalised velocity‐time signals between session one, session two and session three (A). Mean and SD normalised velocity‐time signals for CMJ_AEL_ in session one versus two, session two versus three and session one versus three, respectively, are presented in (B–D). The solid vertical lines represent the dumbbell release point, and the dashed vertical lines represent the lowest position.

**FIGURE 5:**
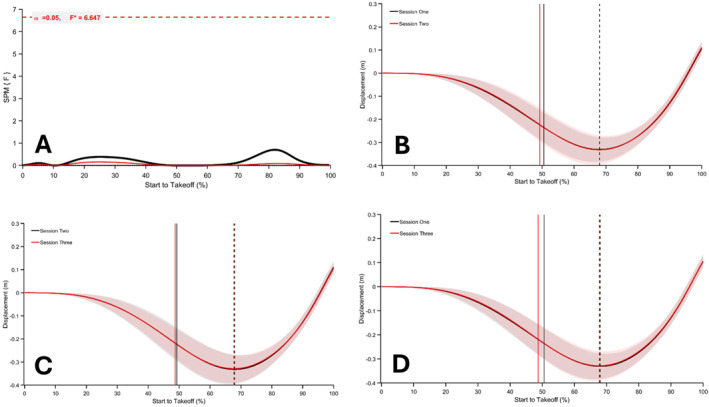
SPM repeated measures ANOVA during CMJ_AEL20_ comparing normalised displacement‐time signals between session one, session two and session three (A). Mean and SD normalised velocity‐time signals for CMJ_AEL_ in session one versus two, session two versus three and session one versus three, respectively, are presented in B–D The solid vertical lines represent the dumbbell release point, and the dashed vertical lines represent the lowest position.

The SPM repeated measures ANOVA revealed a significant main effect of session on normalised force‐time data (*p* < 0.05; F* = 6.990; Figure 2). The analysis indicates that force was significantly greater around and after the dumbbell release point, as well as near take‐off, in session one compared to session two (50%–95% of movement time; Figure 3A,B), and from 47% to 48% of movement when session three was compared to session two (*p* < 0.05; Figure 3C,D). Normalised force‐time data from session three were significantly greater between 66% and 96% of the movement time compared to session one (*p* < 0.001; Figure 3E,F). Results from the SPM repeated measures ANOVA on the normalised velocity‐time and displacement‐time data did not reveal any significant between session differences (*p* > 0.05; Figures 4 and 5).

## 4 Discussion

The primary aim of this study was to investigate the effects of familiarisation on CMJ_AEL20_ in youth athletes by comparing normalised force‐, velocity‐ and displacement‐time data between three testing sessions. A secondary purpose was to examine the reliability, agreement and differences between discrete CMJ_AEL20_ variables. Supporting our first hypothesis, the SPM analysis revealed a significant effect of session for normalised force‐time comparisons, with differences identified between session one to two and one to three (Figure 3). There were also significant differences between session two and three; however, this was limited to approximately 1% of the movement (Figure 3). The normalised velocity‐time and displacement‐time data comparisons did not reveal any significant effects (Figures 4 and 5). There were also no significant differences between CMJ_AEL20_ discrete variables, which supports our second hypothesis (Table 4). The reliability data reveal that other than braking and unweighting time, the remaining variables met an acceptable level of between‐session reliability (Table 2). Only unweighting vGRF%, braking and propulsion mean vGRF and propulsion mean velocity met an acceptable level of within‐session reliability (Table 3). Furthermore, the mean bias between sessions was good for all variables (≤ 10%), although fixed bias was identified in braking mean vGRF and propulsion mean velocity (session one vs. two and two vs. three, respectively). Good to acceptable limits of agreement (≤ 20%) were observed for jump height, braking mean vGRF, propulsion mean vGRF and propulsion mean velocity (Figure 1; Supporting Information S1: Appendix A), with proportional bias observed for unweighting vGRF% (session two vs. three and one vs. three), braking mean vGRF (session two vs. three) and braking mean velocity (session one vs. two).

There were significant session‐to‐session differences in normalised force‐time data, whereas normalised velocity‐ and displacement‐time data remained consistent. Importantly, no significant differences were observed prior to the dumbbell release point, suggesting participants maintained a consistent force application strategy during this part of the movement. This period primarily includes the unweighting phase, which is initiated by relaxation or activation of lower limb musculature to allow the hip and knee joints to flex under the effects of gravity which facilitates the development of negative kinetic energy (McMahon et al. 2018; Harry et al. 2019). Previously, researchers have identified effective unweighting as critical for optimising subsequent braking and propulsion performance, albeit during unloaded CMJs (Krzyszkowski et al. 2022; McHugh et al. 2021). For example, McHugh et al. (2021) reported that athletes who jumped higher were more effective at unweighting, reducing their vGRF to around 19% of body weight in approximately 0.38 s. In the present study, participants reduced their vGRF to approximately 45% of system weight in 0.50 s, which likely reflects a more conservative unweighting strategy due to the added dumbbell mass. Specifically, performing the unweighting phase rapidly would result in participants having to exert more force in order to decelerate and brake their downward motion during the countermovement (Krzyszkowski et al. 2022). Although it was not quantified in the current investigation, the participants may have lacked the eccentric strength necessary to perform the countermovement rapidly (Nishiumi et al. 2023; L. A. Bridgeman et al. 2018); however, this is also difficult to understand with the analysis of only one load. Nonetheless, recent recommendations suggest that relatively weaker individuals with less training experience and AEL familiarity should be able to tolerate and benefit from loads around 20% of body mass (J. Merrigan et al. 2022). Furthermore, the greatest acute improvements have been observed following the application of AEL between 15% and 30% of body mass during PJT (Lloyd et al. 2022; Aboodarda et al. 2013; Godwin et al. 2021; Aboodarda et al. 2014). Therefore, a consideration for muscular strength and a range of loads should be included in future CMJ_AEL_ studies in youth athletes.

Significant differences in normalised force‐time data between session one and two and one and three were apparent between 50% to 95% and 66%–96% of movement time, respectively. In session one and three, the mean portion of the force‐time signal exhibited a bimodal force application strategy (i.e., a single vGRF peak) compared to a unimodal strategy (i.e., two distinct vGRF peaks) in session two. The practical relevance of these different force‐time curve shapes is unclear as there is inconsistent evidence for their relationship to CMJ performance. For example, some authors propose that a bimodal curve is associated with enhanced braking and propulsion performance (Peng et al. 2019), whereas others suggest that it represents inefficient utilisation of the stretch‐shortening cycle (Kennedy and Drake 2018). Nonetheless, the bimodal shape seen in session one and three may represent a more effective proximal‐to‐distal sequence whereby the proximal joints begin extending prior to the distal joints (Peng et al. 2019; Cefai et al. 2024). More specifically, it is possible that the addition of dumbbells and subsequent loading of the trunk and hip joint enhances the initial contribution of the hip joint (Cefai et al. 2024). Equally, the bimodal shape of the force‐time curve and particularly the fact that the second peak was higher than the first may suggest an increased acceleration of the hip joint shortly before take‐off (Augste and Grauer 2024). However, further investigation is required with consideration for joint level mechanics to understand individual contributions of the hip, knee and ankle. Taken together, these findings suggest that a bimodal force application strategy may be preferred in youth athletes performing CMJ_AEL20_ after a period of three familiarisation sessions.

Differences observed in the shape of the force‐time data between sessions may also be expected to result in changes in discrete performance variables (Cormie et al. 2009; McMahon et al. 2017). However, there were no differences between sessions and minimal bias in the current study. First, this underlines the importance of considering the entire movement rather than solely assessing discrete performance variables. Second, these findings align with previous studies that have investigated the influence of familiarisation on loaded and unloaded CMJ performance (Cabarkapa et al. 2024; Moir et al. 2004, 2005; Nibali et al. 2015). It is also possible that in the process of familiarising with CMJ_AEL20_, the participants in this study utilised different movement strategies to achieve a similar outcome. This emphasises the value of implementing instructions in future research, as the current investigation adopted traditional unloaded CMJ instructions that may not have been appropriate during CMJ_AEL_. Given that the unweighting and braking phase include the external load and the propulsion phase does not, future CMJ_AEL_ studies should consider phase specific cues, for example, ‘*unweight rapidly and brake hard*’ (Handford et al. 2022).

Previous studies have sought to understand the reliability of PJT as a means of implementing it in practice to understand developmental trends (Bright et al. 2023), training adaptations (Berton et al. 2018), acute changes in neuromuscular fatigue (Hughes et al. 2022) and readiness to train (Murr et al. 2023). The CMJ was selected to incorporate AEL in this investigation owing to its excellent within‐ and between‐session reliability in similar populations (Bright et al. 2023; Meylan et al. 2012). However, the purpose of this study was not to quantify reliability to assess the suitability of CMJ_AEL_ as a monitoring tool, rather, it was to understand if participants in this study could be familiarised with the movement within three testing sessions. After adopting recent recommendations for analysing CMJ_AEL_ (Bright et al. 2024), our results indicate that unweighting vGRF%, braking mean vGRF and propulsion mean vGRF showed excellent absolute and moderate to excellent relative reliability within all sessions. Furthermore, aside from braking phase time, all other discrete variables demonstrated acceptable between‐session reliability. The mean bias results also suggest that all CMJ_AEL20_ variables display good between‐session agreement (bias ≤ 10%); however, the LOA was only good to acceptable for jump height, braking mean vGRF, propulsion mean vGRF and propulsion mean velocity (≤ 20%; Figure 1). The current findings also suggests that unweighting and braking phase variables contain particularly large random errors (LOA > 20%). Previous studies have noted that the downward phase of a CMJ tends to be more variable in children and adolescents, before it transitions to a more stable pattern through growth and maturation (Bright et al. 2023). As a result of holding and releasing dumbbells, it is possible that the participants in this study experienced even greater variability during this phase of CMJ_AEL20_. For example, across all three testing sessions, participants released the dumbbells anywhere from 0.01 to 0.47 s prior to their lowest countermovement position. For some, this release occurred during the unweighting phase, which may have shortened braking phase time due to an abrupt decrease in system mass. Conversely, participants who held the dumbbells throughout most of the braking phase would have encountered more time to decelerate the CoM in preparation for propulsion. As such, future research should consider if instructional cues are helpful in stabilising the dumbbell release point relative to participants’ lowest countermovement position and how this impacts unweighting and braking phase characteristics using the analytical methods applied in this study (Bright et al. 2024).

Although the current study adopted predefined thresholds based upon a consideration of familiarisation for training rather than formal monitoring, it is recommended that Bland–Altman analyses are supplemented with the evaluation of both fixed and proportional biases (Ludbrook 2010; Giavarina 2015). Fixed bias was identified in two CMJ_AEL20_ variables, with braking mean vGRF decreasing between session one and two and propulsion mean velocity increasing between session two and three (Figure 1 and Supporting Information S1: Appendix A; *p* < 0.05). This suggests that participants adapted between sessions one and two in how they decelerated their CoM, likely employing a more cautious strategy to cope with the handling and release of dumbbells, as previously noted. Regarding propulsion mean velocity, it appears that between sessions two and three, participants became better able to utilise the increased braking demands associated with CMJ_AEL20_ and more effectively translate them into the propulsion phase. Proportional bias was identified in unweighting vGRF%, braking mean vGRF and braking mean velocity (Supporting Information S1: Appendix A), indicating that between session differences varied systematically with the magnitude of the measured values. For example, in unweighting vGRF%, proportional bias was observed in both session one versus three (*β* = −0.26, adjusted *R* ^2^ = 0.05) and session two versus three (*β* = −0.33, adjusted *R* ^2^ = 0.17), indicating that participants with higher unweighting vGRF% values (i.e., less effective unweighting) exhibited greater between‐session variability (e.g., inconsistent negative acceleration or hesitation at the movement onset). Similarly, braking mean vGRF showed proportional bias between sessions two and three (*β* = −0.09, adjusted *R* ^2^ = 0.04) and braking mean velocity between sessions one and two (*β* = 0.22, adjusted *R* ^2^ = 0.06), again highlighting that differences were not constant across the measurement range (e.g., varying control or intensity during the braking phase). Together, these patterns suggest that individuals with lesser or more inconsistent CMJ_AEL20_ performance, particularly in early movement phases, may show more variable responses between sessions.

The current study has limitations that need to be considered when interpreting the results. First, the protocol only considered CMJ_AEL20_; however, it may be hypothesised that familiarisation and associated learning effects would be different when adopting other dumbbell loads (e.g., 30%–40% of body mass). Second, although participants were instructed to release the dumbbells as close to their lowest countermovement position as possible, variations in the exact release point could have influenced the braking and unweighting phase characteristics, which were shown to be more variable between sessions. This variability, especially in braking time, may also be influenced by individual differences in physical development beyond the PHV categorisation, as younger athletes generally exhibit greater movement variability. Lastly, technical difficulties in maintaining consistent force application strategies throughout the movement may have affected some of the observed results, suggesting that participants may require further familiarisation sessions to stabilise their performance.

In summary, the participants in this study demonstrated significant session‐to‐session differences in force production during CMJ_AEL20_, particularly after the release of dumbbells. Familiarisation with the movement improved the reliability of most discrete performance variables; however, unweighting and braking phase variables showed the greatest variability. Therefore, it is possible that the participants in this study would have benefitted from additional sessions to stabilise this part of the CMJ_AEL_; nonetheless, propulsion phase variables were reliable from session one. Taken together, these findings highlight the importance of providing adequate time dedicated to practicing the CMJ and technique associated with releasing the dumbbells when incorporating CMJ_AEL_ into youth S&C programmes.

## Conflicts of Interest

The authors declare no conflicts of interest.

## Supporting information


Supporting Information S1
