# Supplementary material for: The Effects of Familiarisation on Countermovement Jumps with Handheld Dumbbell Accentuated Eccentric Loading in Youth Athletes
Source: Eur J Sport Sci. 2025 Aug 12;25(9):e70033. doi: 10.1002/ejsc.70033 (PMC12342230; doi:10.1002/ejsc.70033)
Supplement: Supplementary file 1 — Supporting Information S1 [file EJSC-25-e70033-s001.docx]

**APPENDIX A**


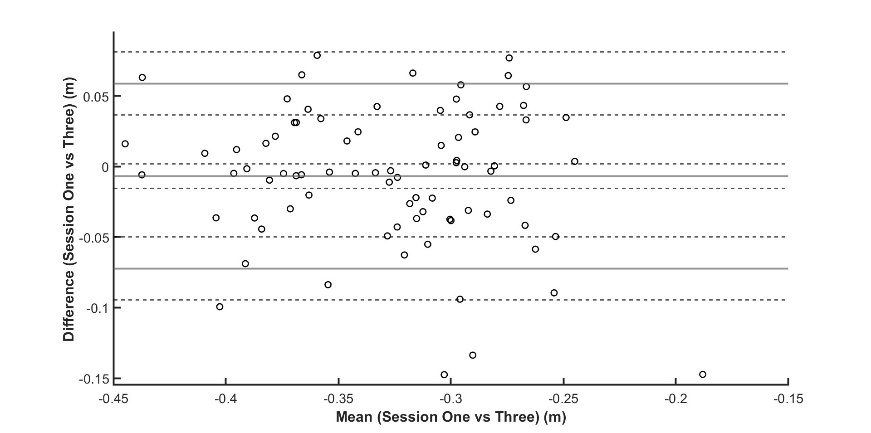

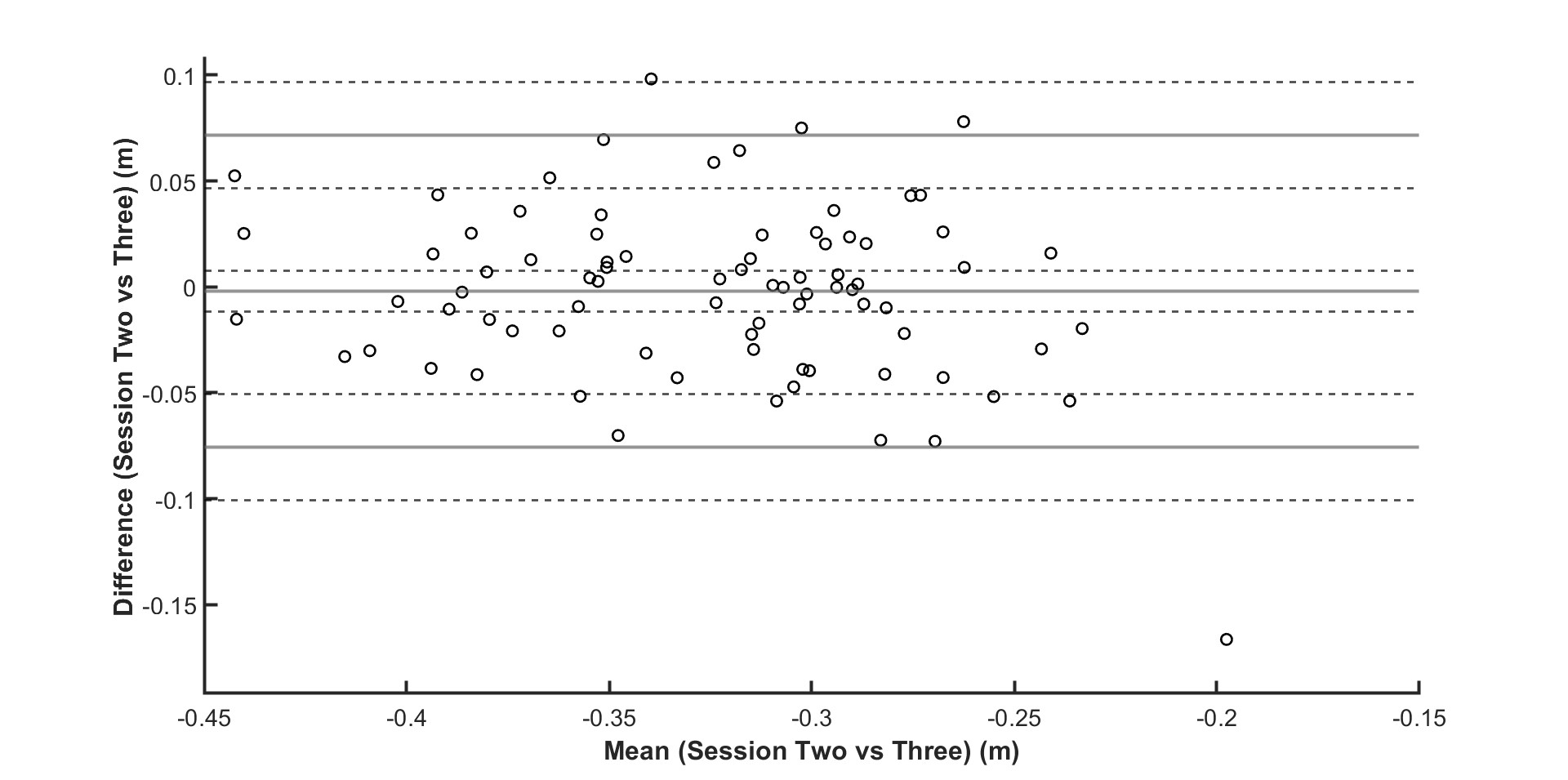

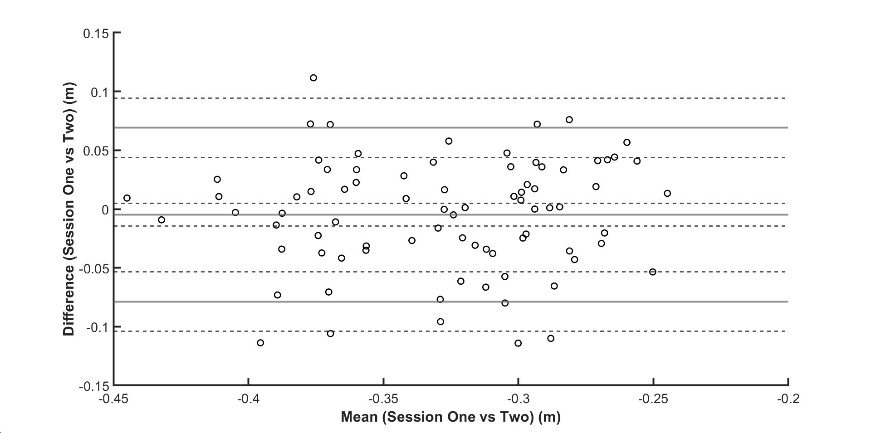

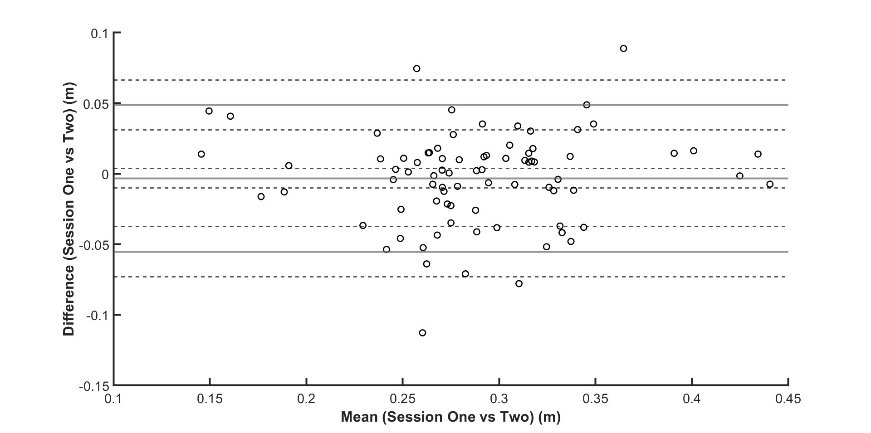

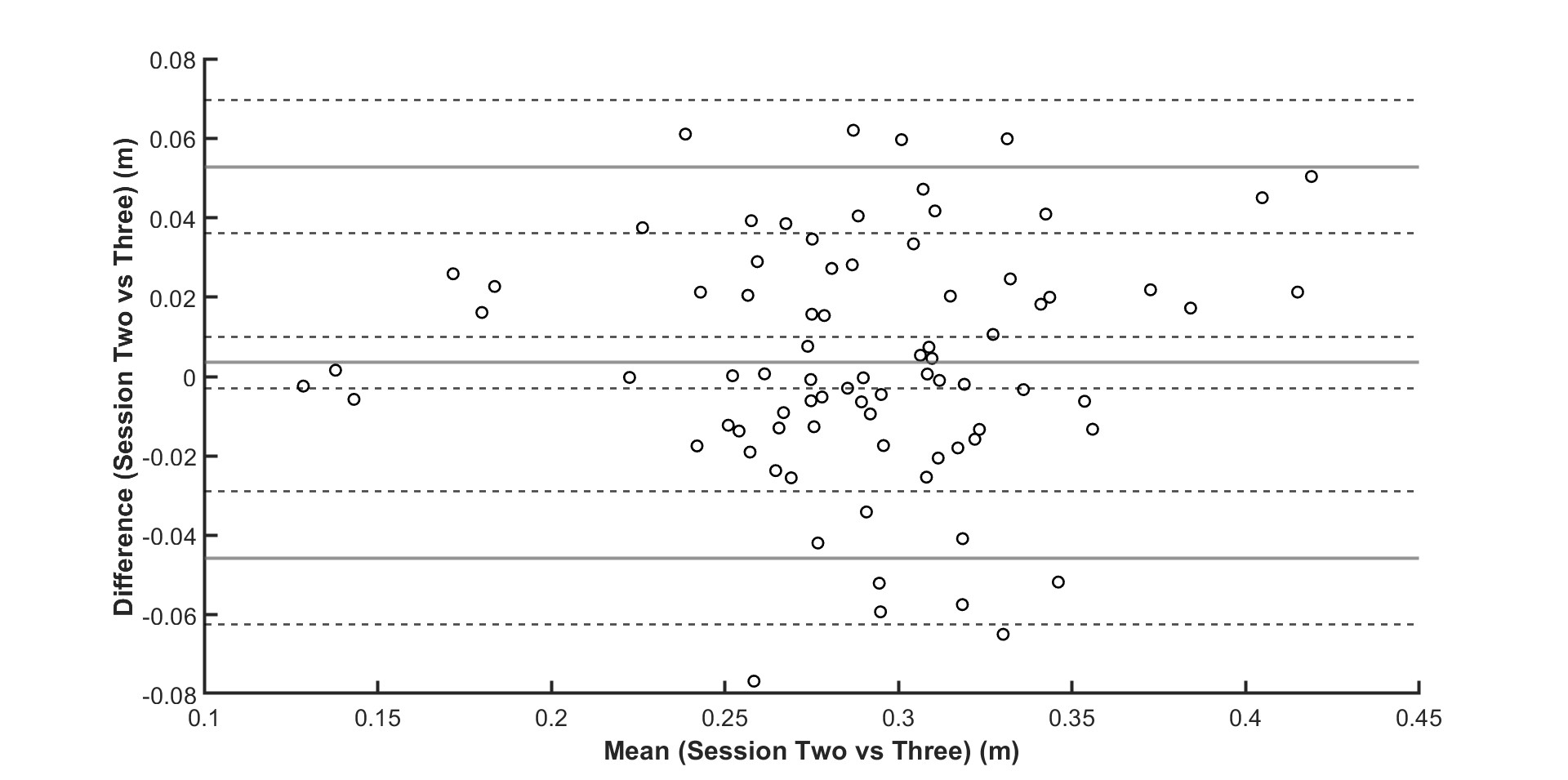

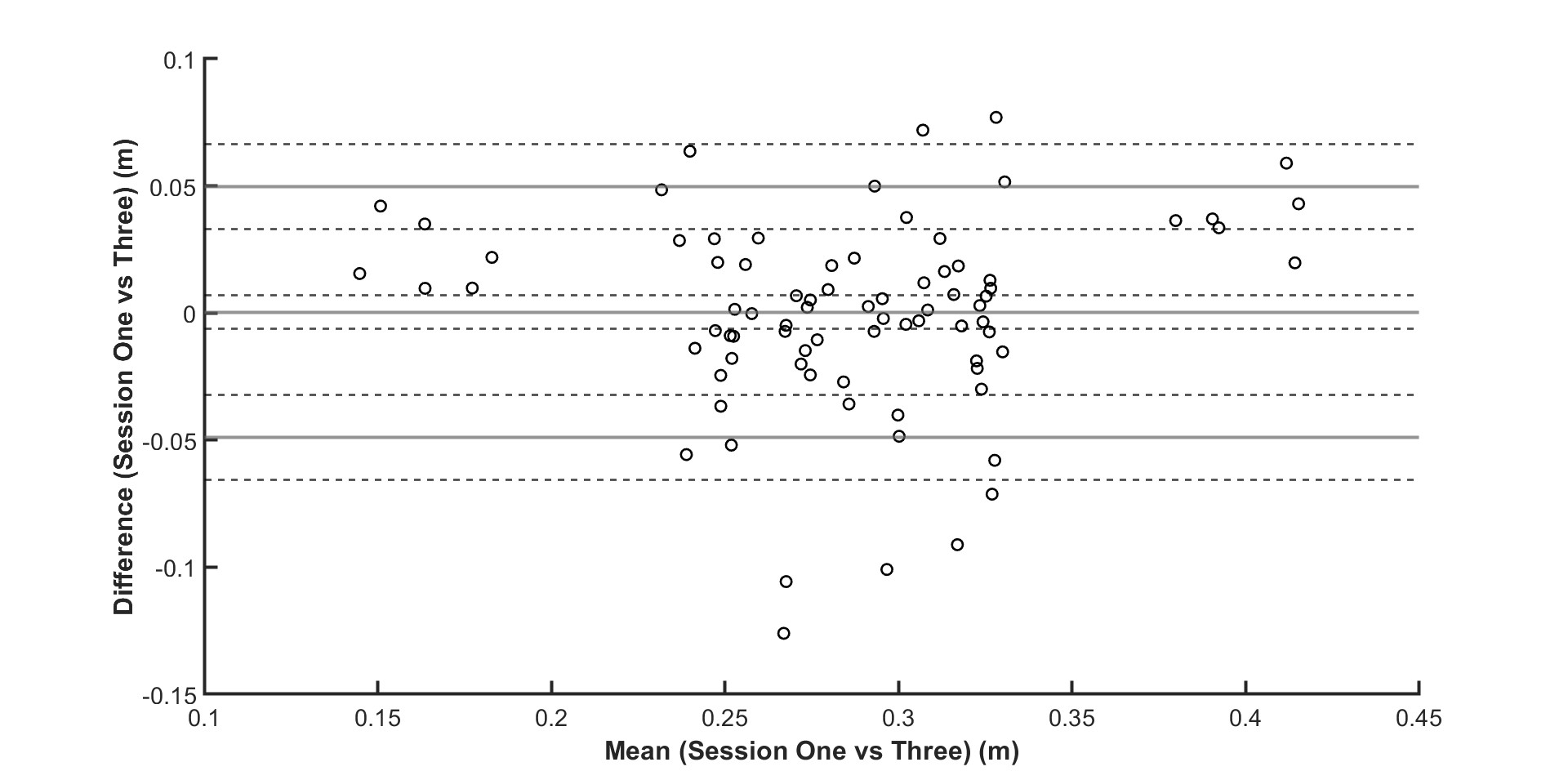


**A**

**B**

**C**

**D**

**E**

**F**

Upper LOA = 0.049 m

(CI_95_: 0.031, 0.067)

Bias = -0.003 m

(CI_95_: -0.010, 0.004)

Lower LOA = -0.055 m

(CI_95_: -0.073, -0.038)

Upper LOA = 0.053 m

(CI_95_: 0.036, 0.070)

Bias = 0.004 m

(CI_95_: -0.003, 0.010)

Lower LOA = -0.046 m

(CI_95_: -0.062, -0.029)

Upper LOA = 0.054 m

(CI_95_: 0.036, 0.072)

Bias = 0.000 m

(CI_95_: -0.007, 0.007)

Lower LOA = -0.053 m

(CI_95_: -0.071, -0.035)

Upper LOA = 0.069 m

(CI_95_: 0.044, 0.094)

Bias = -0.005 m

(CI_95_: -0.015, 0.005)

Lower LOA = -0.079 m

(CI_95_: -0.104, -0.054)

Upper LOA = 0.072 m

(CI_95_: 0.047, 0.097)

Bias = -0.002 m

(CI_95_: -0.012, 0.008)

Lower LOA = -0.076 m

(CI_95_: -0.101, -0.050)

Upper LOA = 0.059 m

(CI_95_: 0.036, 0.081)

Bias = -0.007 m

(CI_95_: -0.015, 0.002)

Lower LOA = -0.072 m

(CI_95_: -0.095, -0.050)

**Figure 1.** Repeated measures Bland-Altman assessment of agreement between session one vs two, session two vs three and session one vs three. Note: Figures A, B and C illustrate the plots for jump height. Figures D, E and F illustrate the plots for countermovement depth. The solid grey lines represent the bias and upper and lower limits of agreement (LOA). The dashed black lines represent the 95% confidence intervals for the bias and LOA.

**Figure 2.** Repeated measures Bland-Altman assessment of agreement between session one vs two, session two vs three and session one vs three. Note: Figures A, B and C illustrate the plots for unweighting phase time. Figures D, E and F illustrate the plots for braking phase time. Figures G, H and I illustrate the plots for propulsion phase time. The solid grey lines represent the bias and upper and lower limits of agreement (LOA). The dashed black lines represent the 95% confidence intervals for the bias and LOA.


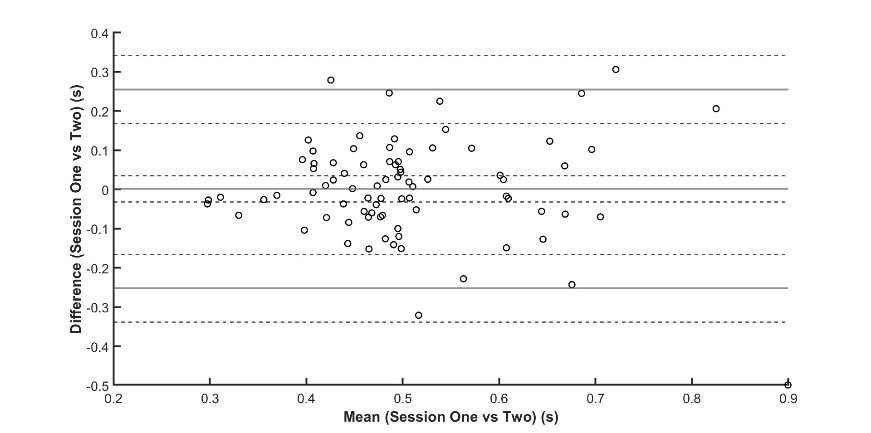

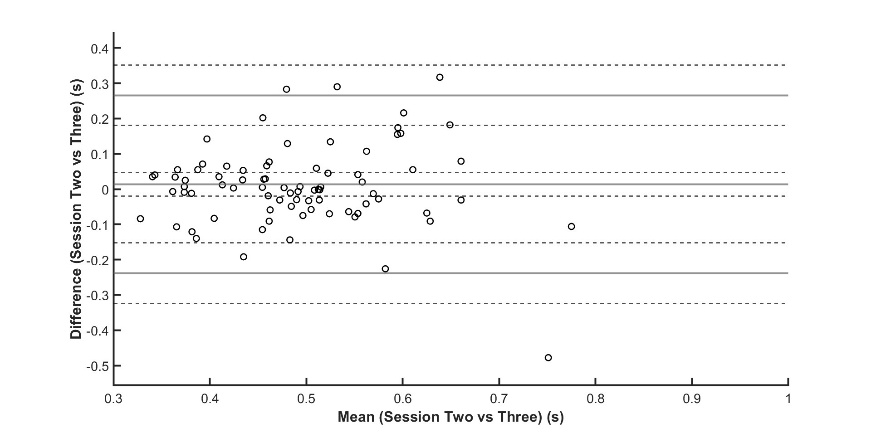

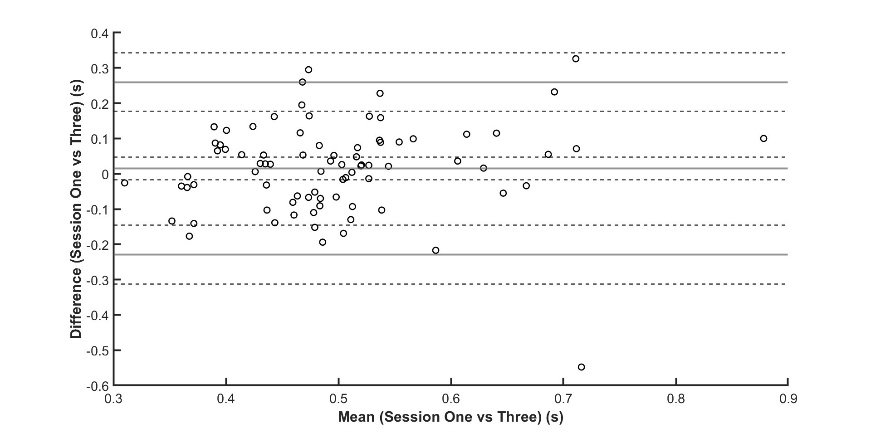

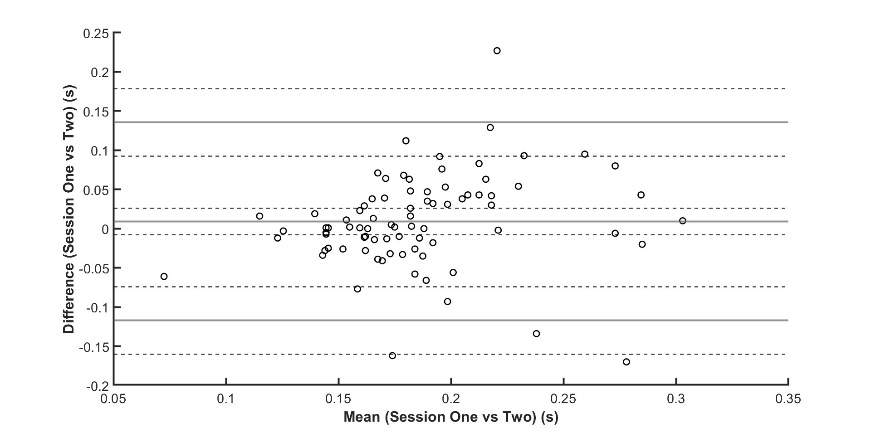

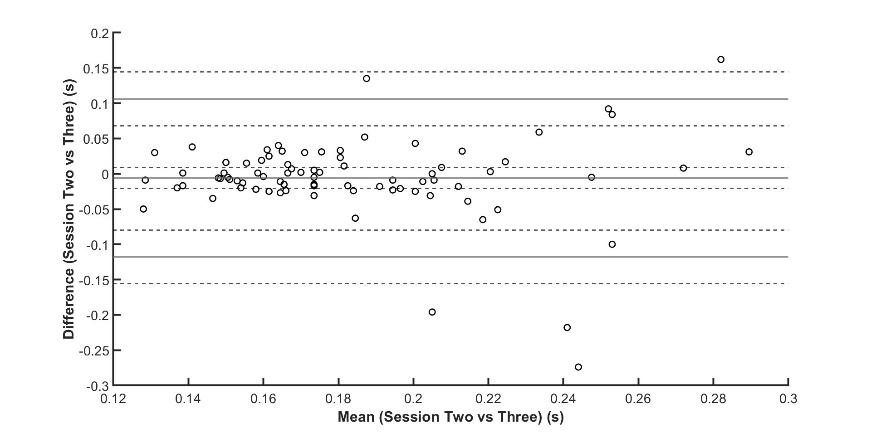

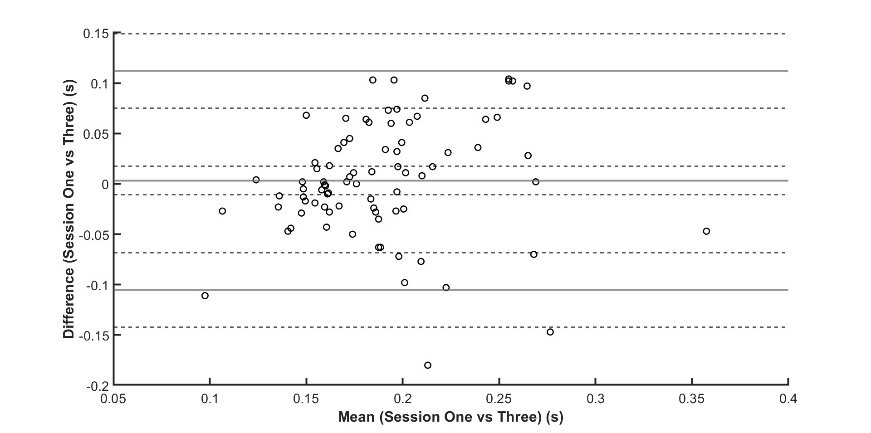

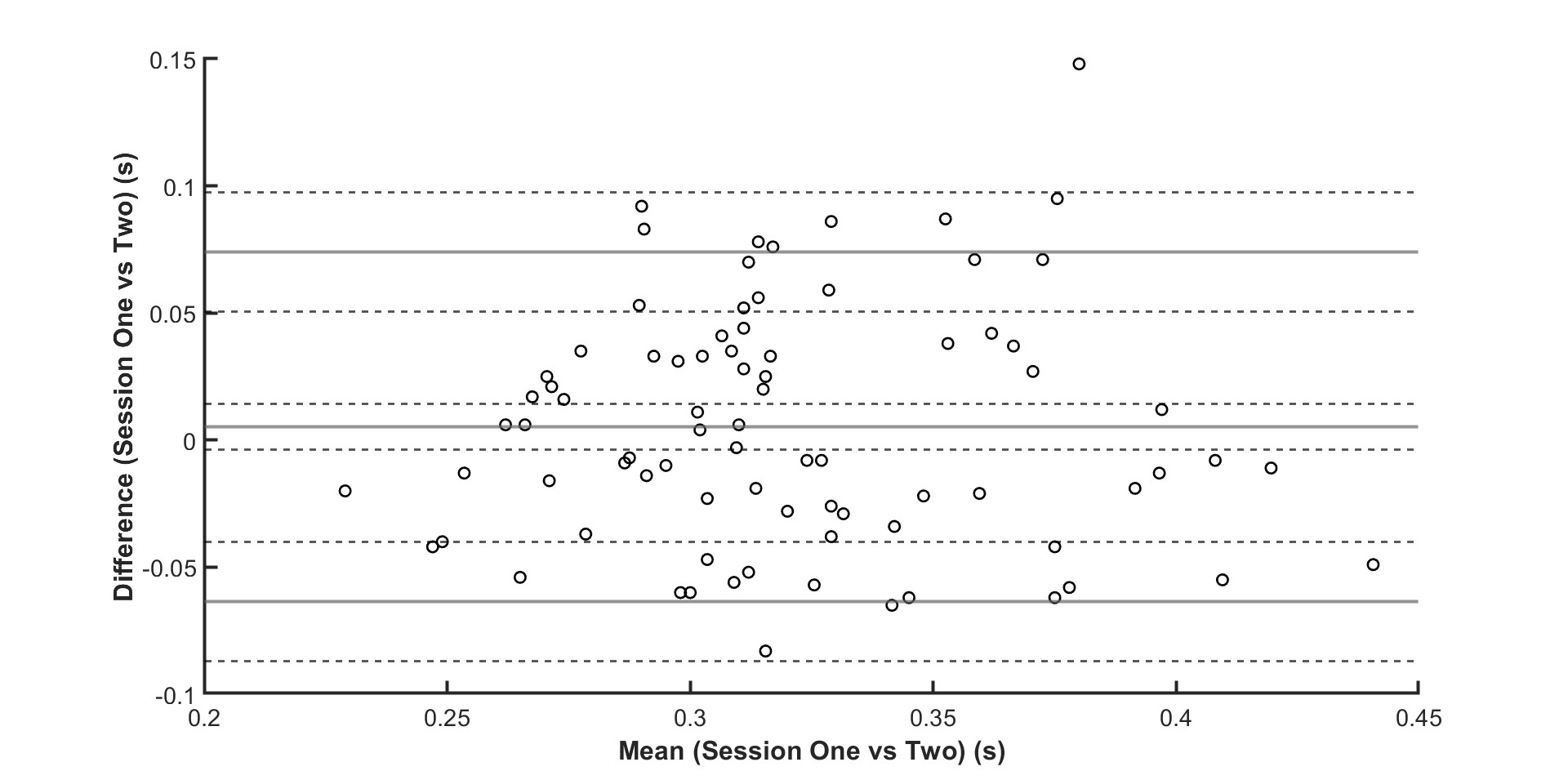

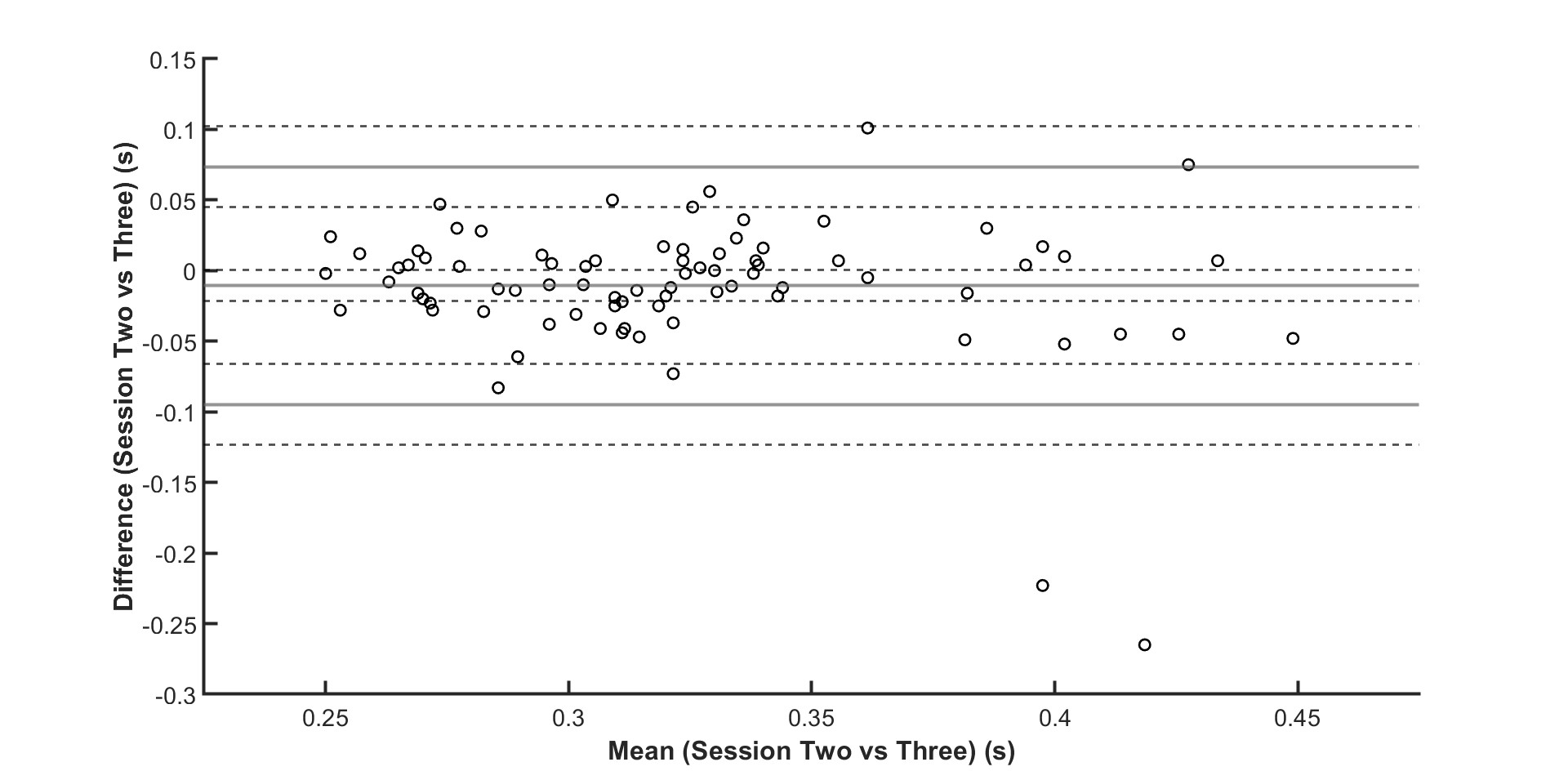

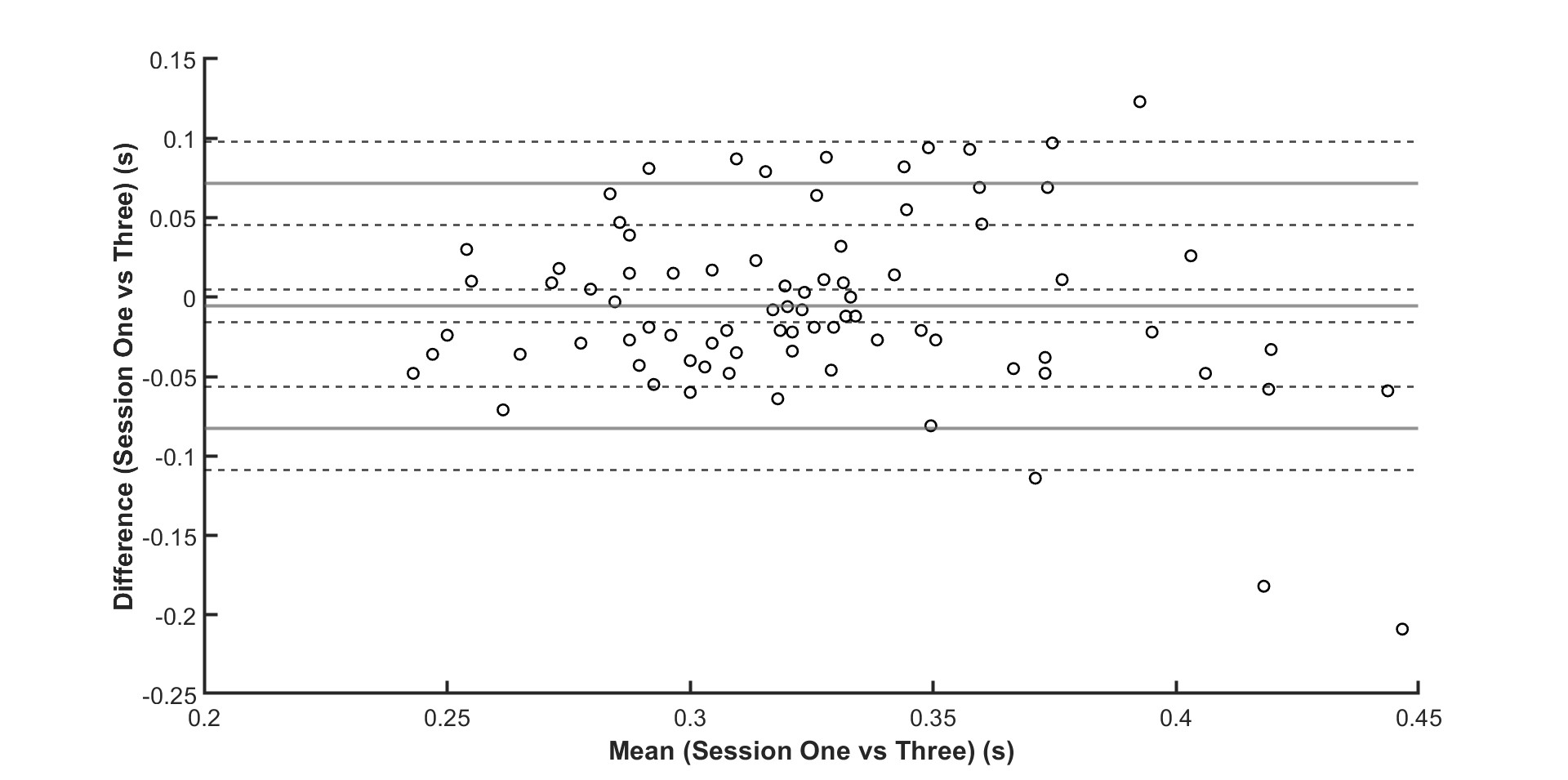


**A**

**B**

**C**

**D**

**E**

**F**

**G**

**H**

**I**

Upper LOA = 0.255 s

(CI_95_: 0.169, 0.341)

Bias = 0.001 s

(CI_95_: -0.032, 0.035)

Lower LOA = -0.252 s

(CI_95_: -0.338, -0.166)

Upper LOA = 0.266 s

(CI_95_: 0.180, 0.352)

Bias = 0.014 s

(CI_95_: -0.020, 0.047)

Lower LOA = -0.238 s

(CI_95_: -0.324, -0.153)

Upper LOA = 0.260 s

(CI_95_: 0.176, 0.343)

Bias = 0.015 s

(CI_95_: -0.017, 0.047)

Lower LOA = -0.229 s

(CI_95_: -0.313, -0.146)

Upper LOA = 0.106 s

(CI_95_: 0.068, 0.144)

Bias = -0.006 s

(CI_95_: -0.021, 0.009)

Lower LOA = -0.118 s

(CI_95_: -0.156, -0.080)

Upper LOA = 0.106 s

(CI_95_: 0.068, 0.144)

Bias = -0.006 s

(CI_95_: -0.021, 0.009)

Lower LOA = -0.118 s

(CI_95_: -0.156, -0.080)

Upper LOA = 0.136 s

(CI_95_: 0.093, 0.179)

Bias = 0.009 s

(CI_95_: -0.007, 0.026)

Lower LOA = -0.117 s

(CI_95_: -0.160, -0.074)

Upper LOA = 0.074 s

(CI_95_: 0.051, 0.098)

Bias = 0.005 s

(CI_95_: -0.004, 0.014)

Lower LOA = -0.064 s

(CI_95_: -0.087, -0.040)

Upper LOA = 0.073 s

(CI_95_: 0.045, 0.102)

Bias = -0.011 s

(CI_95_: -0.022, 0.000)

Lower LOA = -0.095 s

(CI_95_: -0.123, -0.066)

Upper LOA = 0.072 s

(CI_95_: 0.045, 0.098)

Bias = -0.005 s

(CI_95_: -0.016, 0.005)

Lower LOA = -0.082 s

(CI_95_: -0.109, -0.056)


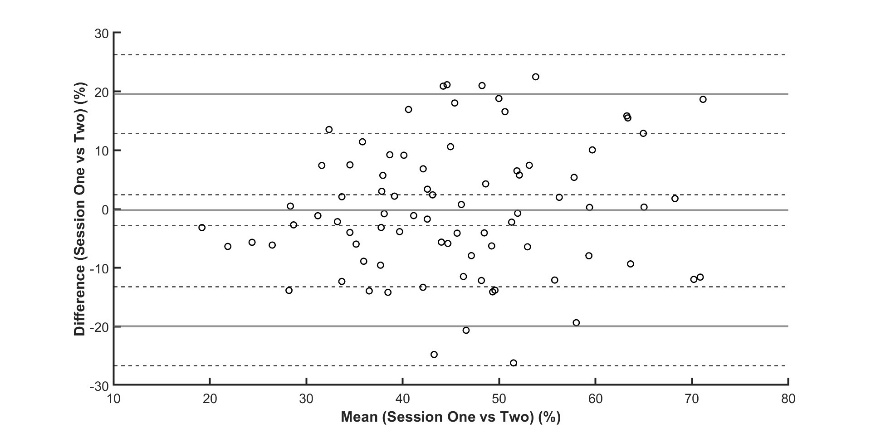

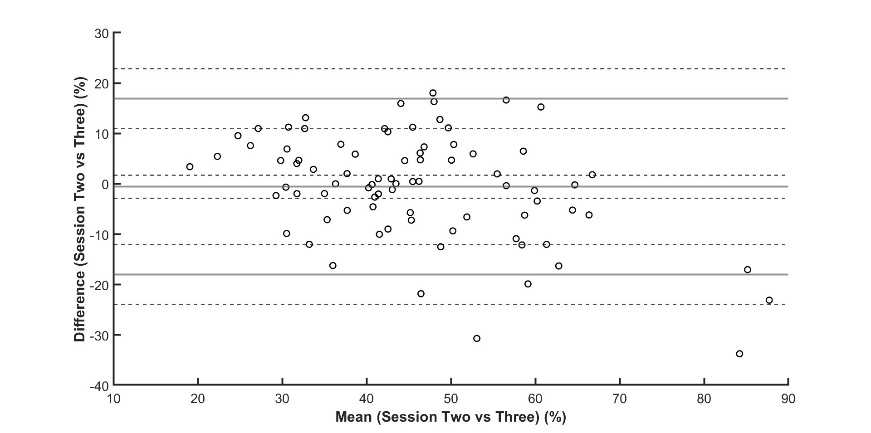

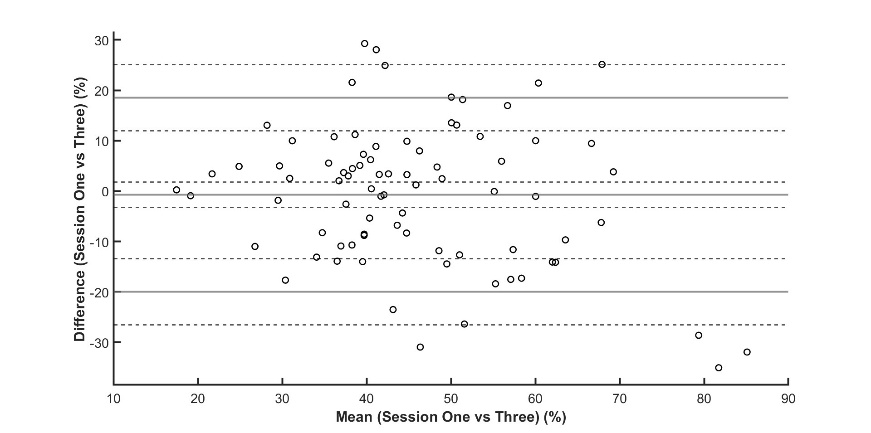

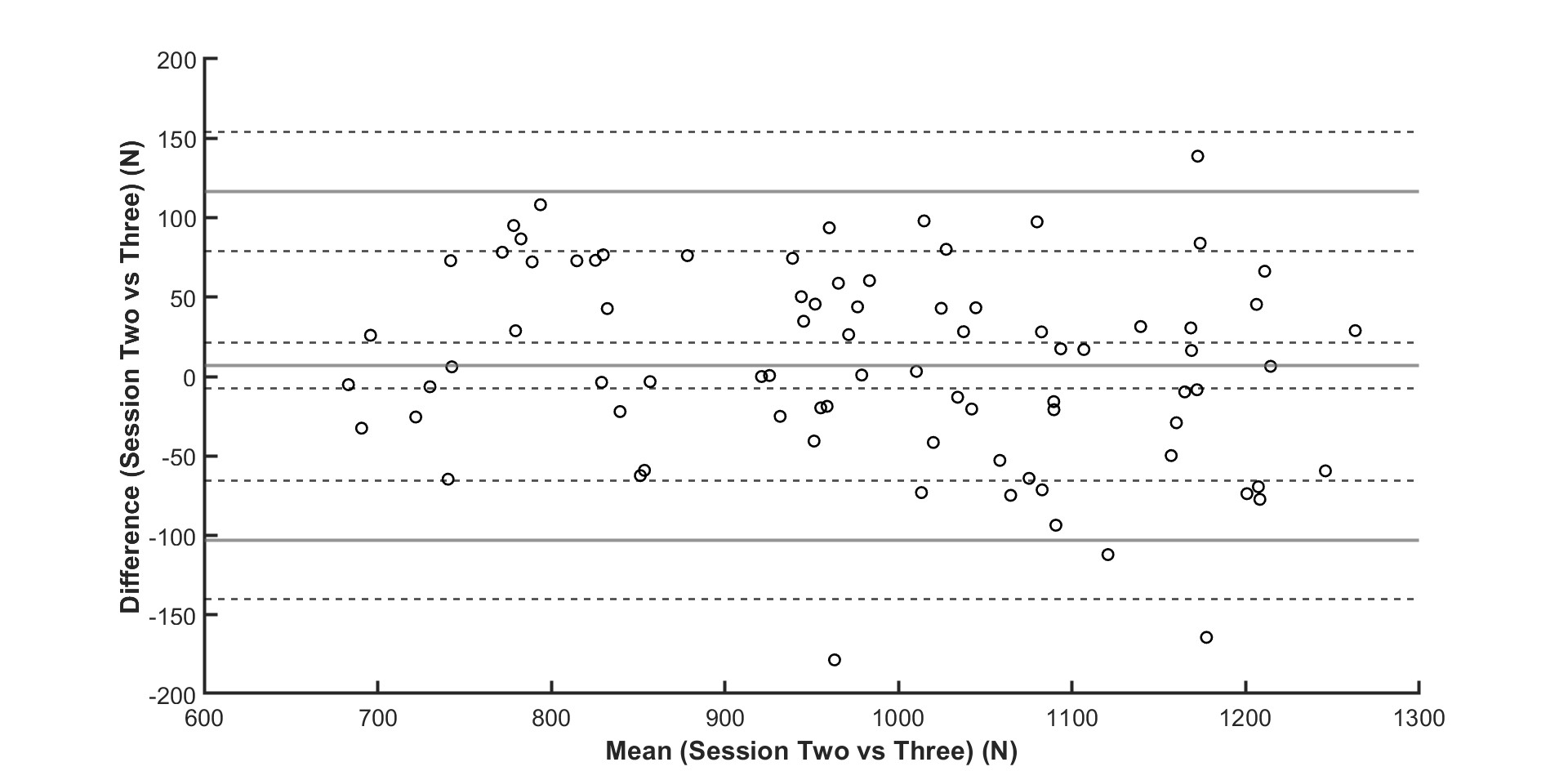

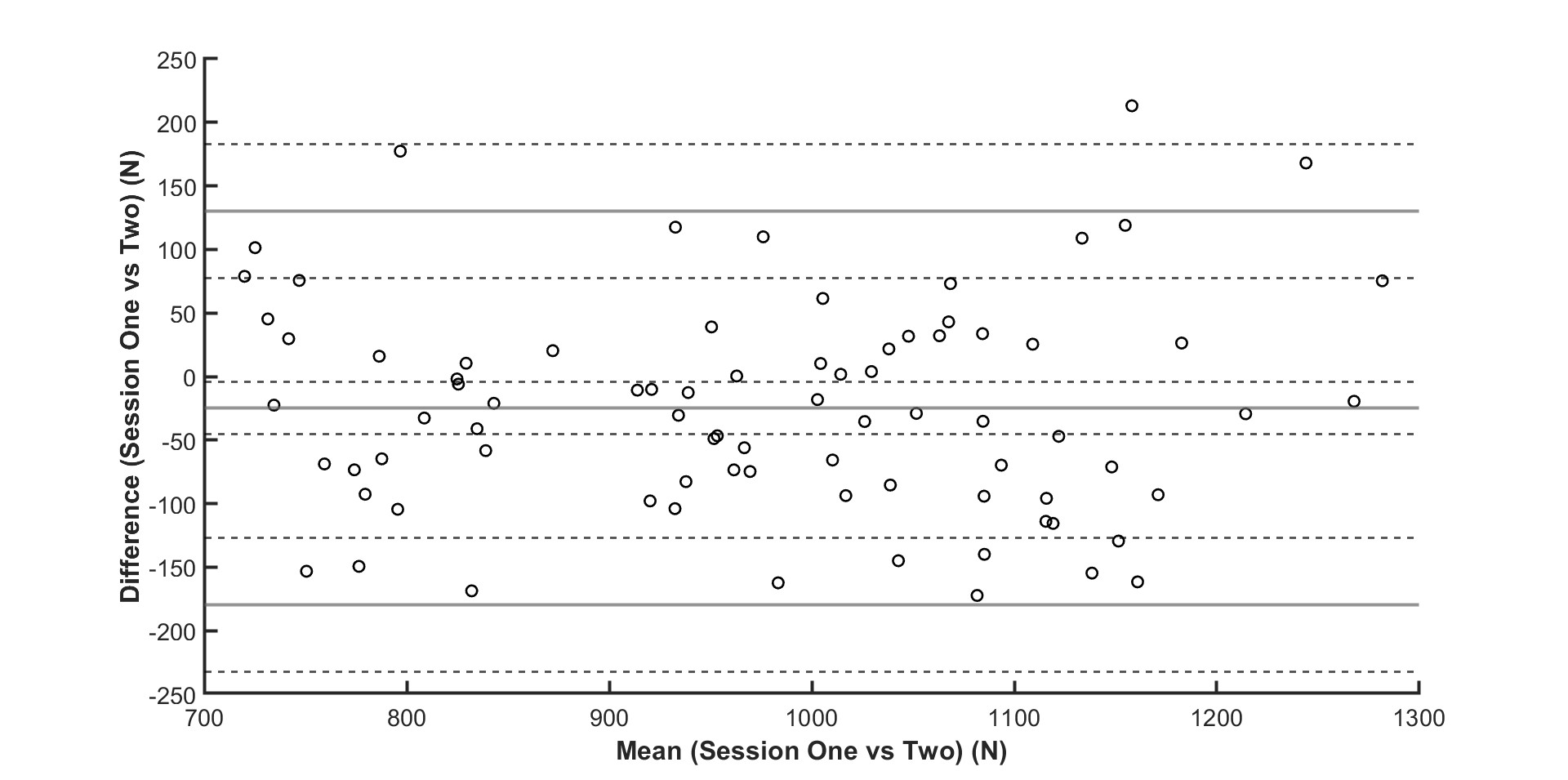

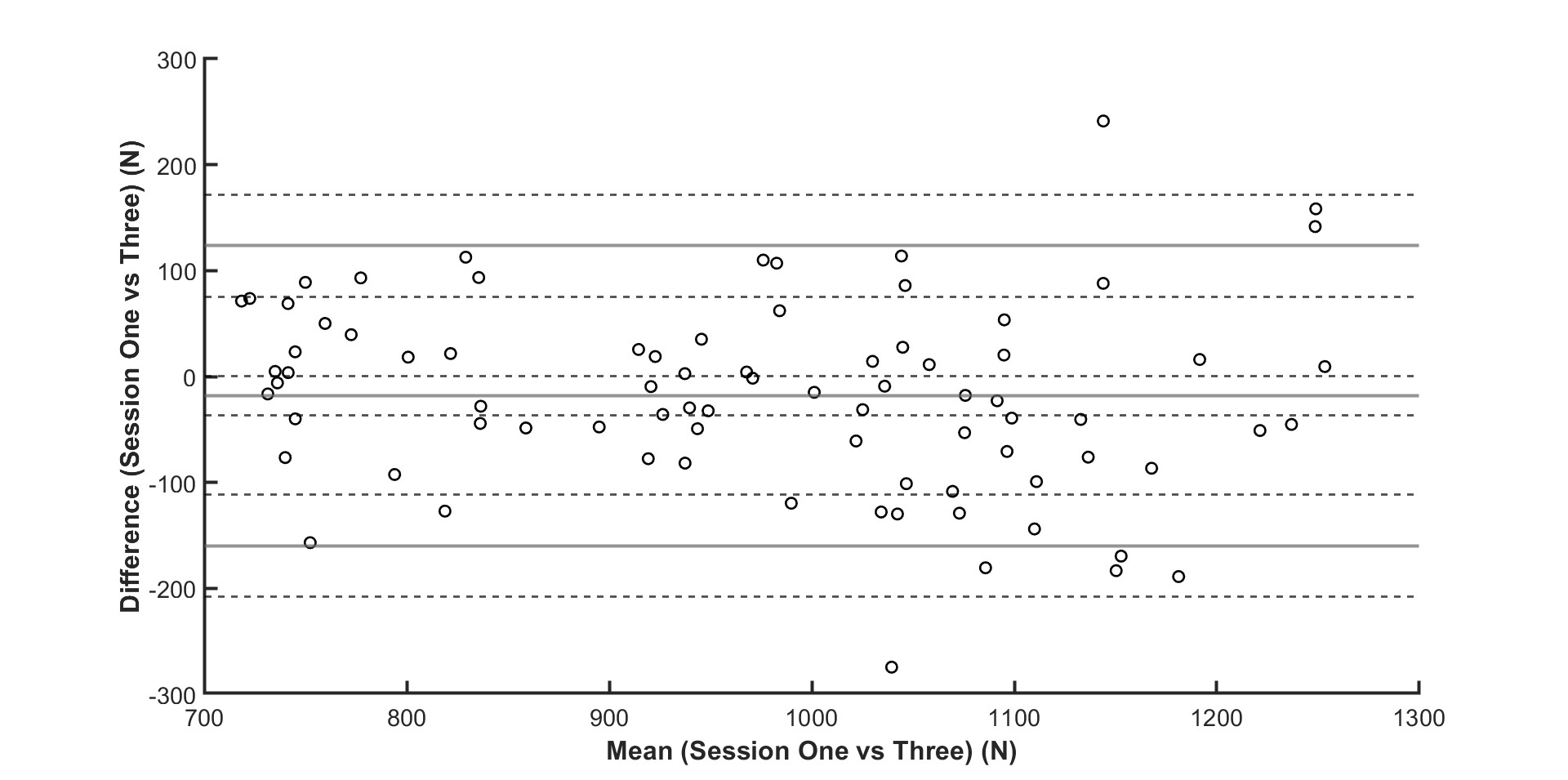

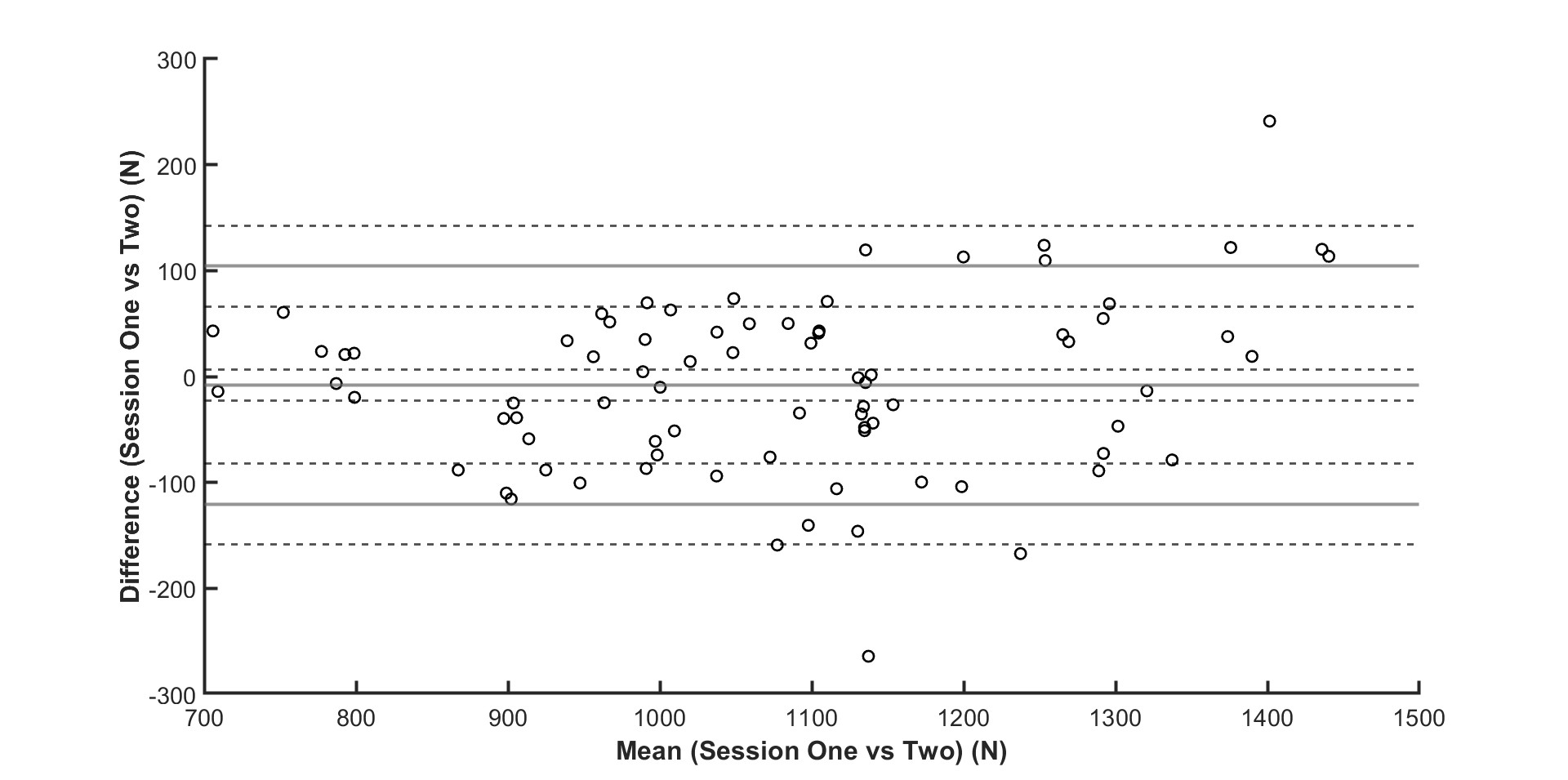

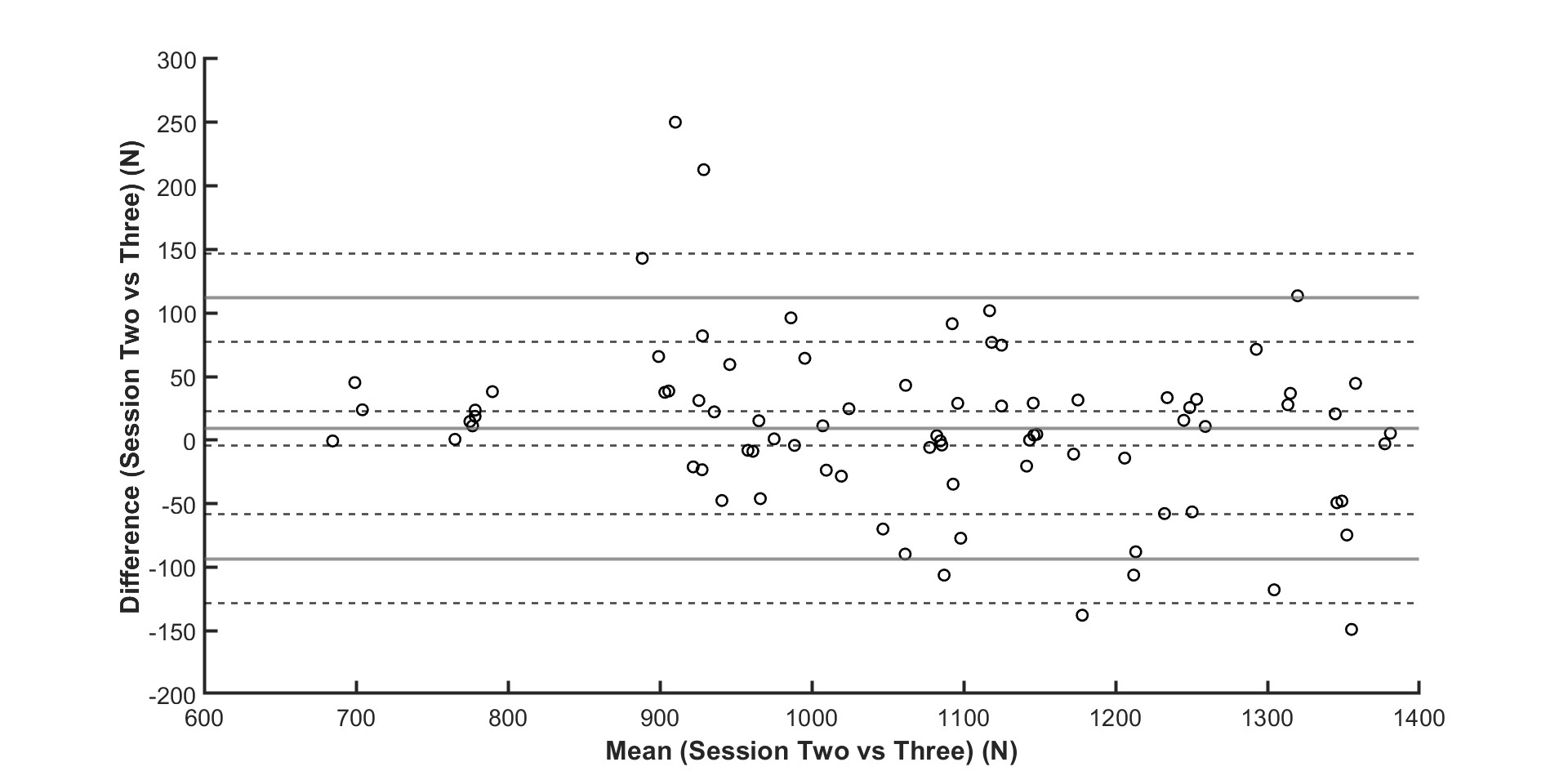

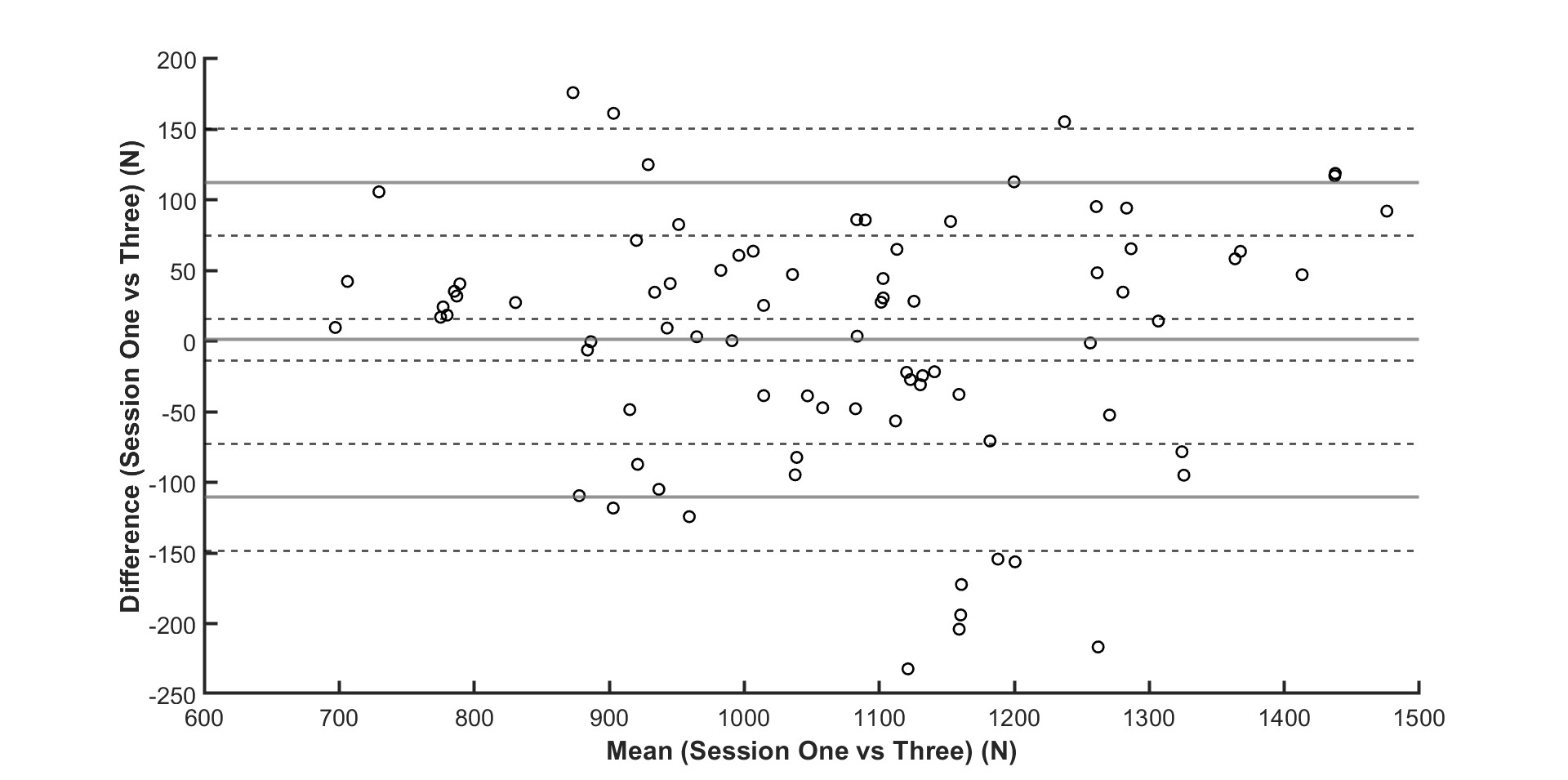


**A**

**B**

**C**

**D**

**E**

**F**

**G**

**H**

**I**

Upper LOA = 19.57 %

(CI_95_: 12.85, 26.29)

Bias = -0.18 %

(CI_95_: -2.78, 2.43)

Lower LOA = -19.92 %

(CI_95_: -26,64, -13.20)

Upper LOA = 16.91 %

(CI_95_: 10.96, 22.85)

Bias = -0.56 %

(CI_95_: -2.86, 1.74)

Lower LOA = -18.03 %

(CI_95_: -23.97, -12.08)

Upper LOA = 18.52 %

(CI_95_: 11.97, 25.08)

Bias = -0.74 %

(CI_95_: -3.27, 1.80)

Lower LOA = -19.99 %

(CI_95_: -26.55, -13.44)

Upper LOA = 123.54 N

(CI_95_: 75.34, 171.74)

Bias = -18.10 N

(CI_95_: -36.77, 0.56)

Lower LOA = -159.75 N

(CI_95_: -207.95, -111.54)

Upper LOA = 116.47 N

(CI_95_: 79.13, 153.81)

Bias = 6.76 N

(CI_95_: -7.70, 21.22)

Lower LOA = -102.96 N

(CI_95_: -140.29, -65.62)

Upper LOA = 129.95 N

(CI_95_: 77.27, 182.63)

Bias = -24.86 N

(CI_95_: -45.26, -4.46)

Lower LOA = -179.67 N

(CI_95_: -232.35, -126.99)

Upper LOA = 104.17 N

(CI_95_: 65.93, 142.42)

Bias = -8.21 N

(CI_95_: 23.02, 6.60)

Lower LOA = -120.59 N

(CI_95_: -158.84, -82.35)

Upper LOA = 112.00 N

(CI_95_: 77.04, 146.95)

Bias = 9.27 N

(CI_95_: -4.27, 22.81)

Lower LOA = -93.46 N

(CI_95_: -128.42, -58.50)

Upper LOA = 112.62 N

(CI_95_: 74.66, 150.59)

Bias = 1.06 N

(CI_95_: -13.65, 15.76)

Lower LOA = -110.51 N

(CI_95_: -148.48, -72.54)

**Figure 3.** Repeated measures Bland-Altman assessment of agreement between session one vs two, session two vs three and session one vs three. Note: Figures A, B and C illustrate the plots for unweighting vGRF%. Figures D, E and F illustrate the plots for braking mean vGRF. Figures G, H and I illustrate the plots for propulsion mean vGRF. The solid grey lines represent the bias and upper and lower limits of agreement (LOA). The dashed black lines represent the 95% confidence intervals for the bias and LOA.


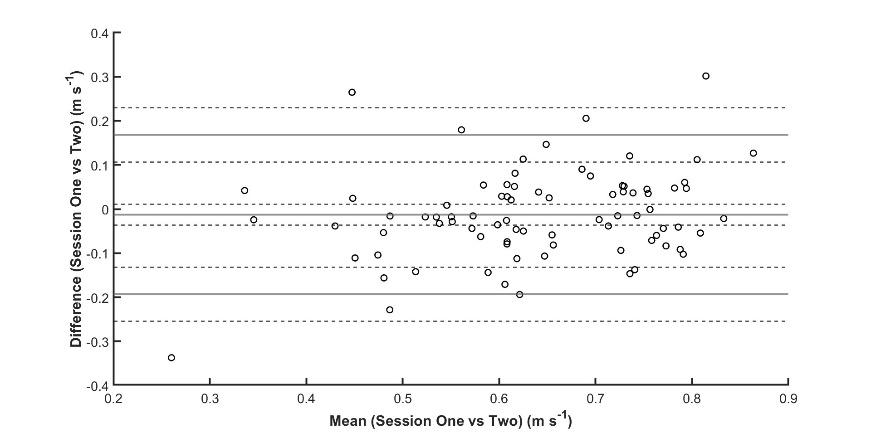

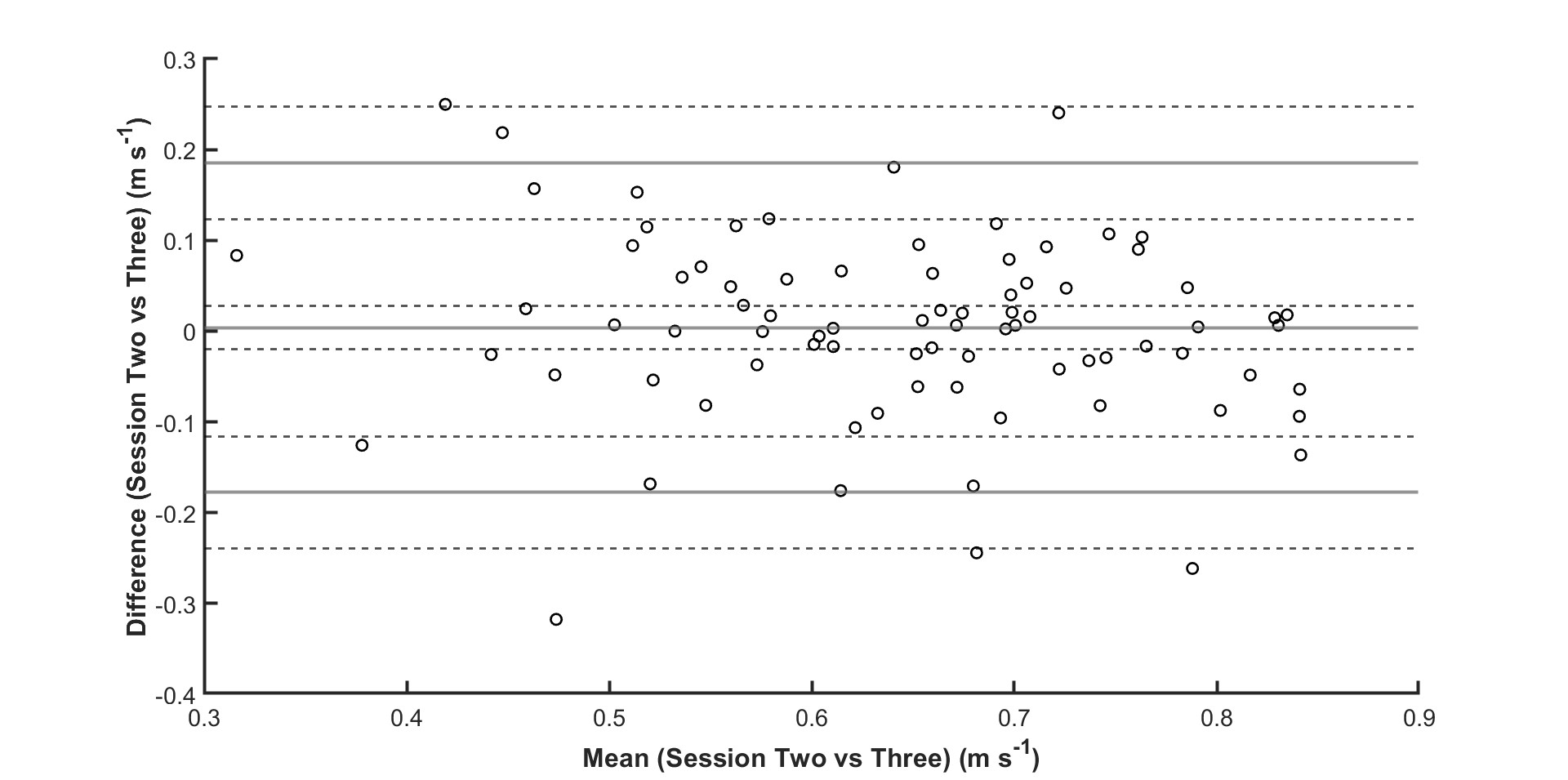

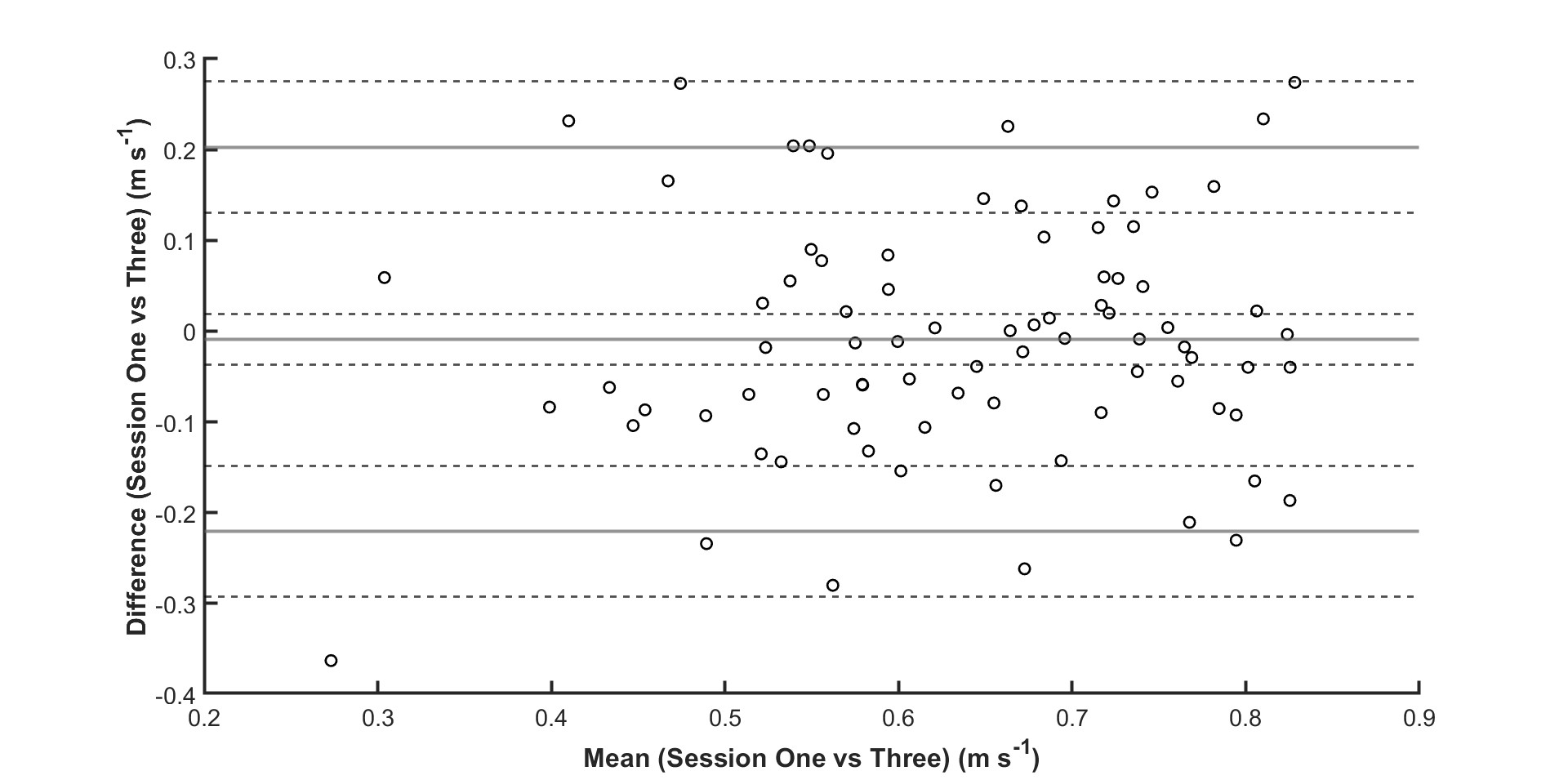

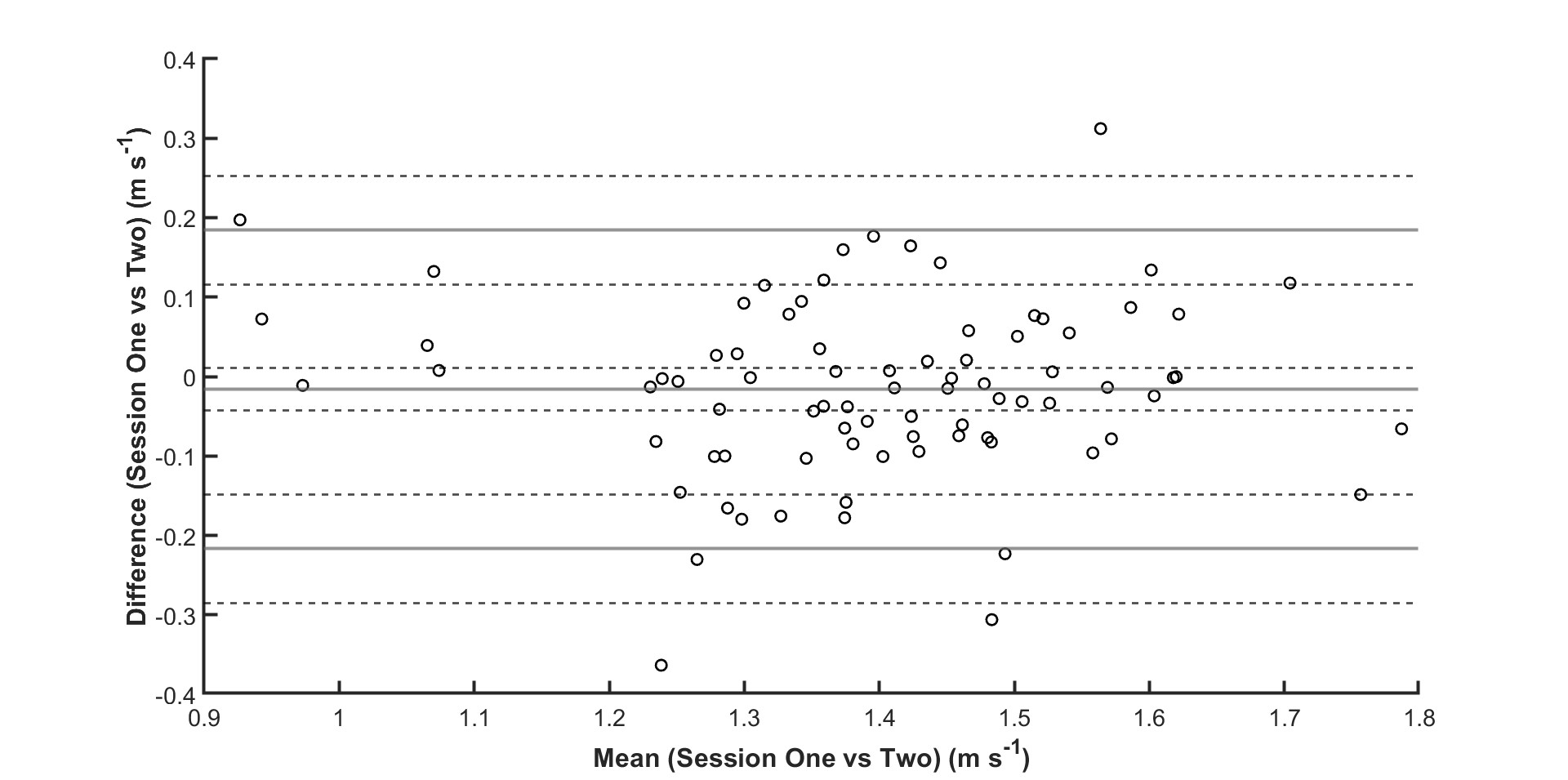

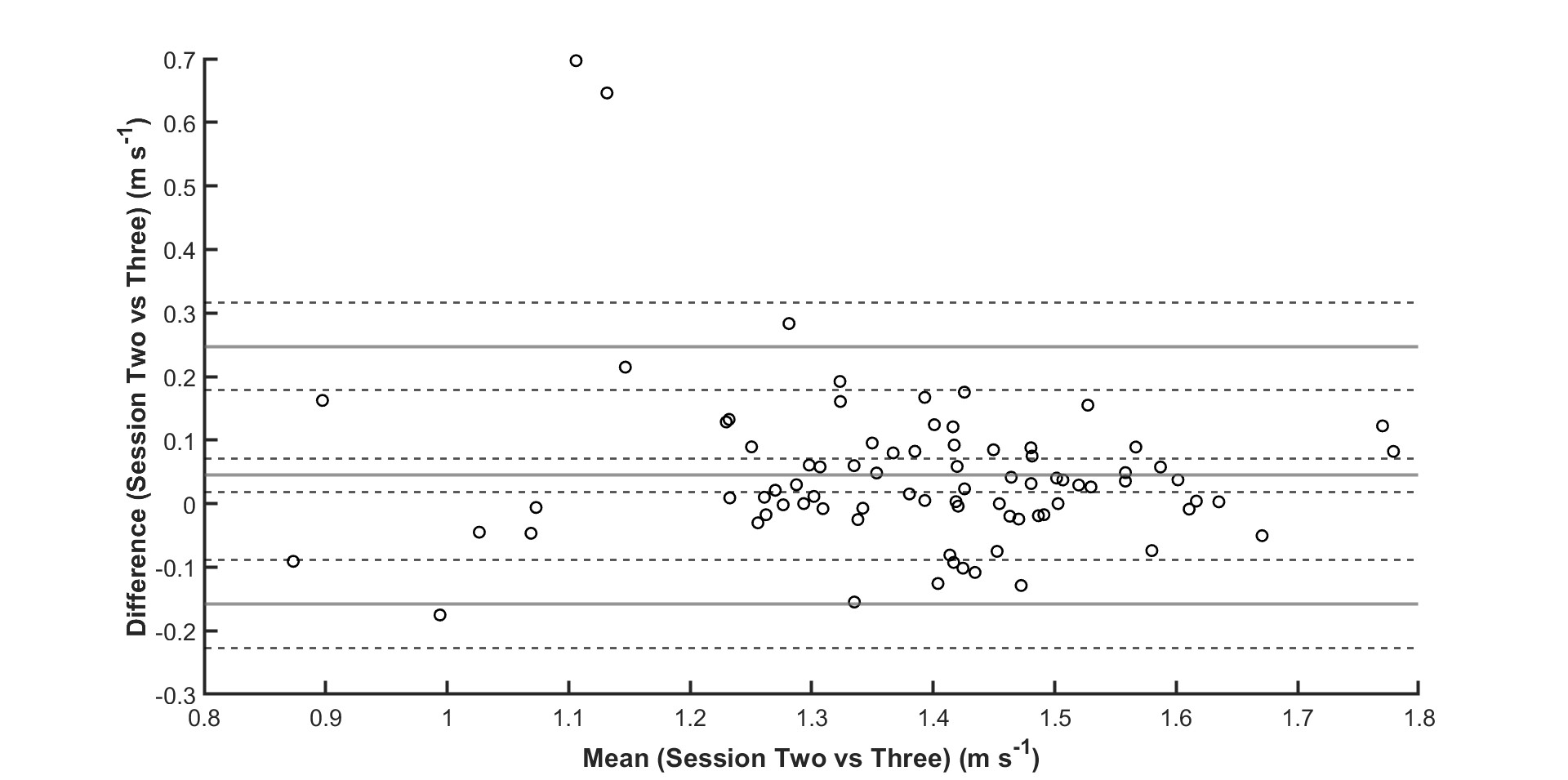

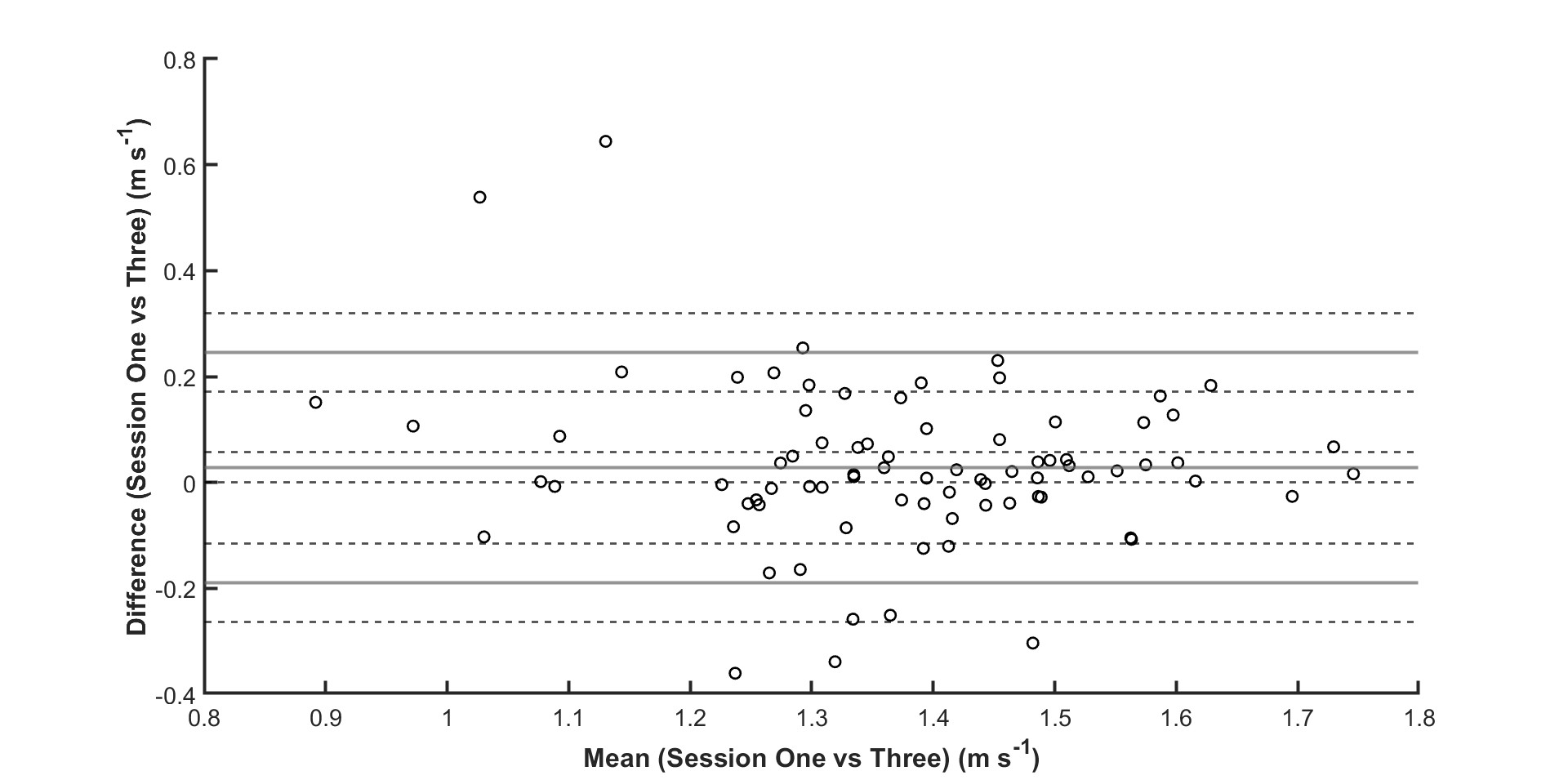


**A**

**B**

**C**

**D**

**E**

**F**

Upper LOA = 0.17 m·s^-1^

(CI_95_: 0.11, 0.23)

Bias = -0.01 m·s^-1^

(CI_95_: -0.04, 0.01)

Lower LOA = -0.19 m·s^-1^

(CI_95_: -0.25, -0.13)

Upper LOA = 0.19 m·s^-1^

(CI_95_: 0.12, 0.25)

Bias = 0.00 m·s^-1^

(CI_95_: -0.02, 0.03)

Lower LOA = -0.18 m·s^-1^

(CI_95_: -0.24, -0.12)

Upper LOA = 0.20 m·s^-1^

(CI_95_: 0.13, 0.27)

Bias = -0.01 m·s^-1^

(CI_95_: -0.04, 0.02)

Lower LOA = -0.22 m·s^-1^

(CI_95_: -0.29, -0.15)

Upper LOA = 0.25 m·s^-1^

(CI_95_: 0.17, 0.32)

Bias = 0.03 m·s^-1^

(CI_95_: 0.00, 0.06)

Lower LOA = -0.19 m·s^-1^

(CI_95_: -0.26, -0.12)

Upper LOA = 0.25 m·s^-1^

(CI_95_: 0.18, 0.32)

Bias = 0.04 m·s^-1^

(CI_95_: 0.02, 0.07)

Lower LOA = -0.16 m·s^-1^

(CI_95_: -0.23, -0.09)

Upper LOA = 0.18 m·s^-1^

(CI_95_: 0.12, 0.25)

Bias = -0.02 m·s^-1^

(CI_95_: -0.04, 0.01)

Lower LOA = -0.22 m·s^-1^

(CI_95_: -0.28, -0.15)

**Figure 4.** Repeated measures Bland-Altman assessment of agreement between session one vs two, session two vs three and session one vs three. Note: Figures A, B and C illustrate the plots for braking mean velocity. Figures D, E and F illustrate the plots for propulsion mean velocity. The solid grey lines represent the bias and upper and lower limits of agreement (LOA). The dashed black lines represent the 95% confidence intervals for the bias and LOA.

**Table 1.** Fixed and proportional bias results for CMJ_AEL20_ outcome and temporal variables.

| **Variable** | **Bias Type** | **Session Comparison** | ***p*** | **Slope (β)** | **Adjusted R²** |
| --- | --- | --- | --- | --- | --- |
| **Jump Height (m)** | **Fixed** | 1 vs 2 | 0.387 | - | - |
|  |  | 2 vs 3 | 0.290 | - | - |
|  |  | 1 vs 3 | 0.934 | - | - |
|  | **Proportional** | 1 vs 2 | 0.651 | 0.03 | -0.01 |
|  |  | 2 vs 3 | 0.669 | 0.03 | -0.01 |
|  |  | 1 vs 3 | 0.465 | 0.06 | -0.01 |
| **Countermovement Depth (m)** | **Fixed** | 1 vs 2 | 0.366 | - | - |
|  |  | 2 vs 3 | 0.665 | - | - |
|  |  | 1 vs 3 | 0.213 | - | - |
|  | **Proportional** | 1 vs 2 | 0.685 | 0.05 | -0.01 |
|  |  | 2 vs 3 | 0.051 | -0.17 | 0.04 |
|  |  | 1 vs 3 | 0.203 | -0.14 | 0.01 |
| **Unweighting Time (s)** | **Fixed** | 1 vs 2 | 0.924 | - | - |
|  |  | 2 vs 3 | 0.330 | - | - |
|  |  | 1 vs 3 | 0.296 | - | - |
|  | **Proportional** | 1 vs 2 | 0.544 | -0.08 | -0.01 |
|  |  | 2 vs 3 | 0.151 | 0.19 | 0.01 |
|  |  | 1 vs 3 | 0.425 | 0.12 | 0.00 |
| **Braking Time (s)** | **Fixed** | 1 vs 2 | 0.171 | - | - |
|  |  | 2 vs 3 | 0.372 | - | - |
|  |  | 1 vs 3 | 0.603 | - | - |
|  | **Proportional** | 1 vs 2 | 0.115 | 0.26 | 0.02 |
|  |  | 2 vs 3 | 0.712 | -0.07 | -0.01 |
|  |  | 1 vs 3 | 0.215 | 0.19 | 0.01 |
| **Propulsion Time (s)** | **Fixed** | 1 vs 2 | 0.327 | - | - |
|  |  | 2 vs 3 | 0.053 | - | - |
|  |  | 1 vs 3 | 0.392 | - | - |
|  | **Proportional** | 1 vs 2 | 0.925 | 0.01 | -0.01 |
|  |  | 2 vs 3 | 0.086 | -0.20 | 0.02 |
|  |  | 1 vs 3 | 0.144 | -0.21 | 0.01 |

**Note:** Fixed bias assessed via paired *t*-tests and proportional bias assessed using linear regression of the difference between sessions against the mean. Negative slopes indicate larger between-session differences at lower average values. Adjusted R² represents the proportion of variance in the difference explained by the average value.

**Table 2.** Fixed and proportional bias results for CMJ_AEL20_ kinetic and kinematic variables.

| **Variable** | **Bias Type** | **Comparison** | ***p*** | **Slope (β)** | **Adjusted R²** |
| --- | --- | --- | --- | --- | --- |
| **Unweighting vGRF (% BW)** | **Fixed** | 1 vs 2 | 0.888 | - | - |
|  |  | 2 vs 3 | 0.636 | - | - |
|  |  | 1 vs 3 | 0.636 | - | - |
|  | **Proportional** | 1 vs 2 | 0.316 | 0.11 | 0.00 |
|  |  | **2 vs 3** | **0.000** | **-0.33** | **0.17** |
|  |  | **1 vs 3** | **0.025** | **-0.26** | **0.05** |
| **Braking Mean vGRF (N)** | **Fixed** | **1 vs 2** | **0.010** | - | - |
|  |  | 2 vs 3 | 0.332 | - | - |
|  |  | 1 vs 3 | 0.072 | - | - |
|  | **Proportional** | 1 vs 2 | 0.913 | 0.01 | -0.01 |
|  |  | **2 vs 3** | **0.047** | **-0.09** | **0.04** |
|  |  | 1 vs 3 | 0.193 | -0.08 | 0.01 |
| **Propulsion Mean vGRF (N)** | **Fixed** | 1 vs 2 | 0.367 | - | - |
|  |  | 2 vs 3 | 0.212 | - | - |
|  |  | 1 vs 3 | 0.915 | - | - |
|  | **Proportional** | 1 vs 2 | 0.099 | 0.08 | 0.02 |
|  |  | 2 vs 3 | 0.013 | -0.10 | 0.06 |
|  |  | 1 vs 3 | 0.737 | -0.02 | -0.01 |
| **Braking Mean Velocity (m·s^-1^)** | **Fixed** | 1 vs 2 | 0.027 | - | - |
|  |  | 2 vs 3 | 0.737 | - | - |
|  |  | 1 vs 3 | 0.547 | - | - |
|  | **Proportional** | **1 vs 2** | **0.014** | **0.22** | **0.06** |
|  |  | 2 vs 3 | 0.126 | -0.15 | 0.02 |
|  |  | 1 vs 3 | 0.465 | 0.09 | -0.01 |
| **Propulsion Mean Velocity (m·s^-1^)** | **Fixed** | 1 vs 2 | 0.202 | - | - |
|  |  | **2 vs 3** | **0.003** | - | - |
|  |  | 1 vs 3 | 0.099 | - | - |
|  | **Proportional** | 1 vs 2 | 0.958 | 0.00 | -0.01 |
|  |  | 2 vs 3 | 0.140 | -0.13 | 0.02 |
|  |  | 1 vs 3 | 0.180 | -0.14 | 0.01 |

**Note:** Fixed bias assessed via paired *t*-tests. Proportional bias assessed using linear regression of the difference between sessions against the mean. Bolded *p*-values and orange shading indicate statistical significance (*p* < 0.05). Unweighting vGRF% refers to the minimum vertical ground reaction force during the unweighting phase, expressed as a percentage of body weight. Negative slopes indicate increasing between-session differences at lower average values. Adjusted R² represents the proportion of variance in the difference explained by the average value.
